# The Moral Choice Machine

**DOI:** 10.3389/frai.2020.00036

**Published:** 2020-05-20

**Authors:** Patrick Schramowski, Cigdem Turan, Sophie Jentzsch, Constantin Rothkopf, Kristian Kersting

**Affiliations:** ^1^Department of Computer Science, Darmstadt University of Technology, Darmstadt, Germany; ^2^German Aerospace Center (DLR), Institute for Software Technology, Cologne, Germany; ^3^Institute of Psychology, Darmstadt University of Technology, Darmstadt, Germany; ^4^Centre for Cognitive Science, Darmstadt University of Technology, Darmstadt, Germany

**Keywords:** moral bias, fairness in machine learning, text-embedding models, natural language processing, AI, machine learning

## Abstract

Allowing machines to choose whether to kill humans would be devastating for world peace and security. But how do we equip machines with the ability to learn ethical or even moral choices? In this study, we show that applying machine learning to human texts can extract deontological ethical reasoning about “right” and “wrong” conduct. We create a template list of prompts and responses, such as “Should I [action]?”, “Is it okay to [action]?”, etc. with corresponding answers of “Yes/no, I should (not).” and "Yes/no, it is (not)." The model's bias score is the difference between the model's score of the positive response (“Yes, I should”) and that of the negative response (“No, I should not”). For a given choice, the model's overall bias score is the mean of the bias scores of all question/answer templates paired with that choice. Specifically, the resulting model, called the Moral Choice Machine (MCM), calculates the bias score on a sentence level using embeddings of the Universal Sentence Encoder since the moral value of an action to be taken depends on its context. It is objectionable to kill living beings, but it is fine to kill time. It is essential to eat, yet one might not eat dirt. It is important to spread information, yet one should not spread misinformation. Our results indicate that text corpora contain recoverable and accurate imprints of our social, ethical and moral choices, even with context information. Actually, training the Moral Choice Machine on different temporal news and book corpora from the year 1510 to 2008/2009 demonstrate the evolution of moral and ethical choices over different time periods for both atomic actions and actions with context information. By training it on different cultural sources such as the Bible and the constitution of different countries, the dynamics of moral choices in culture, including technology are revealed. That is the fact that moral biases can be extracted, quantified, tracked, and compared across cultures and over time.

## 1. Introduction

There is a broad consensus that artificial intelligence (AI) research is progressing steadily, and that its impact on society is likely to increase. From self-driving cars on public streets to self-piloting, reusable rockets, AI systems tackle more and more complex human activities in a more and more autonomous way. This leads to new spheres, where traditional ethics has limited applicability. Both self-driving cars, where mistakes may be life-threatening, and machine classifiers that hurt social matters may serve as examples for entering gray areas in ethics: how does AI embody our value system? Do AI systems learn humanly intuitive correlations? If not, can we contest the AI system?

Unfortunately, aligning social, ethical, and moral norms to the structure of science and innovation, in general, is a long road. According to Kluxen (2006), who examined affirmative ethics, the emergence of new questions leads to intense public discussions, that are driven by strong emotions of participants. And machine ethics (Bostorm and Yudkowsky, 2011; Russell et al., 2015; Kramer et al., 2018) is no exception. Consider, e.g., Caliskan et al.'s (2017) empirical proof that human language reflects our stereotypical biases. Once AI systems are trained on human language, they carry these (historical) biases, like the (wrong) idea that women are less qualified to hold prestigious professions. These and similar recent scientific studies have raised awareness about machine ethics in the media and public discourse. AI systems “have the potential to inherit a very human flaw: bias,” as Socure's CEO Sunil Madhu puts it^1^. AI systems are not neutral with respect to purpose and society anymore. Ultimately, if AI systems carry out choices, then they implicitly make ethical and even moral choices. Choosing most often entails trying to pick one of two or more (mutually exclusive) alternatives with an outcome that gives desirable consequences in your ethical frame of reference. But how do we equip AI systems to make human-like ethical choices?

We start by presenting our previous findings (Jentzsch et al., 2019) with focusing on quantifing deontological ethics, i.e., finding out, whether an action itself is right or wrong. Following Kim and Hooker (2018), for the replication we first focus our attention to atomic actions instead of complex behavioral patterns. Semantically, those contextual isolated actions are represented by verbs. To conduct this assignment, a template list of prompts and responses is created for ethical choices. The template includes questions, such as “Should I kill?,” “Should I love?,” etc. with answer templates “Yes/no, I should (not).” The model's bias score is calculated as the difference between the model's score of the positive response (“Yes, I should”) and that of the negative response (“No, I should not”). For a given choice, the model's overall bias score is the mean of the bias scores of all question/answer templates paired with that choice.

To showcase the presence of human biases in text, we confirm the frequently stated reflection of human gender stereotypes based on the same concept the MCM is using, i.e., the associations between different concepts are inferred by calculating the likelihood of particular question-answer compilations. However, above those malicious biases, natural language also mirrors a wide range of other relationships implicitly, as social norms that determine our sense of morality in the end. Using the MCM, we therefore also demonstrate the presence of ethical valuation in text by generating an ethical bias of actions.

The strong correlation between WEAT values and moral biases at the verb level gives reasons to extend the investigation of the MCM by first inspecting complex human-like choices at the phrase level and second if the MCM can capture a variety of human-like choices reflected by different text-sources. The moral bias of an action is depending on the surrounding context. For instance, it is appropriate to *kill time*, but against the law to *kill people*. Also, since the moral biases imprinted in the text embeddings would depend on the text sources the embeddings trained on, we further investigate the moral biases of complex actions and the changes in moral biases of various corpora. To do so, we first generated a list of context-based actions and collected different datasets such as books published in different centuries, news from the last three decades and constitutions of 193 countries. These newly collected datasets are used to retrain the Universal Sentence Encoder, and to extract the moral biases. Our results show that the MCM is able to capture the moral bias of not just atomic actions but also of actions with surrounding context and one can use this as a tool to extract and examine moral biases across cultural text sources and over time.

This paper is an extension of the conference paper (Jentzsch et al., 2019), where we introduced the basic Moral Choice Machine (MCM). Based on extending Caliskan et al.'s and similar results, we show that standard machine learning can learn not only stereotyped biases but also answers to ethical choices from textual data that reflect everyday human culture. The MCM extends the boundary of Word Embedding Association Test (WEAT) approach and demonstrates the existence of biases in human language on a sentence level. Moreover, accurate imprints of social, ethical and moral choices could be identified. The above-mentioned conference paper, however, considered only atomic actions to evaluate the moral bias enclosed in text embeddings. In this paper, we extend the atomic actions with contextual information which allows us to investigate the moral bias in more detail. We have shown that the MCM not only grasps Do's and Don'ts of the atomic actions but also the changes in moral bias with the contextual information, e.g., kill time has a positive value where kill people has a negative value (the higher the bias, the more acceptable that behavior is). This paper also includes comprehensive experimental results where the Universal Sentence Encoder has been retrained with the text sources of various years and source types, e.g. religious and constitutional documents, books from different centuries, and news from different years. These results are particularly important because we have shown that the characteristics of the retrained model reflect the information that is carried implicitly and explicitly by the source texts. This result changes in the moral bias while the model adapts itself to the given text source.

We proceed as follows: After reviewing our assumptions and the required background, we introduce the MCM and the replication pipeline to rate and rank atomic moral choices. Before concluding, we present our empirical results and the current limitations of the MCM.

## 2. Assumptions and Background

Before describing the MCM, we start by reviewing our assumptions, in particular, what we mean by *moral choices*, and the required background.

### 2.1. Moral Choices

Philosophically, morality referred to the individual's level of “right” and “wrong,” while ethics referred to the “right” and “wrong” arrangements established by a social community. Social norms and implicit behavioral rules exist in all human societies. However, while their presence is omnipresent, they are hardly measurable, or even consistently definable. The underlying mechanisms are still poorly understood. Indeed, any working community has an abstract morale that is essentially valid and must be adhered to. Theoretical concepts, however, have been identified as inconsistent, or even sometimes contradictory. Accordingly, latent ethics and morals have been described as the sum of particular norms which may not necessarily follow logical reasoning. Recently, Lindström et al. (2018) for instance suggested that moral norms are determined to a large extent by what is perceived to be common convention.

Concerning the complexity and intangibility of ethics and morals, we restrict ourselves, as in our previous work (Jentzsch et al., 2019), to a rather basic implementation of this construct, following the theories of deontological ethics. These ask which choices are morally required, forbidden, or permitted instead of asking which kind of a person we should be or which consequences of our actions are to be preferred. Thus, norms are understood as universal rules of what to do and what not to do. Therefore, we focus on the valuation of social acceptance in single verbs to figure out which of them represents a *Do* and which tend to be a *Don't*. Because we specifically chose templates in the first person, i.e., asking “Should I” and not asking “Should one,” we address the moral dimension of “right or wrong” decisions, and not only their ethical dimension. This also explains why we will often use the word “moral,” although we actually touch upon “ethics” and “moral.” To measure the valuation, we make use of implicit association tests (IATs) and their connections to word embeddings.

### 2.2. The Implicit Association Test

The *Implicit Association Test* (IAT) is a well-established tool in social psychology for analyzing attitudes of people without specifically asking for it. This method addresses the issue that people may not always be able or willing to tell what's on their minds, but indirectly reveal it in their behavior. The IAT measures the magnitude of the differential association of contradictory concepts by measuring the decision velocity in an assignment task.

Several investigations in the literature, that are worth mentioning and frequently referred, already use the IAT to identify latent attitudes, including gender and race discrimination. Greenwald et al. (1998) initially introduced the IAT. They found several effects, including both ethically neutral ones, for instance the preference of flowers over insects, and sensitive ones, as the preference of one ethnic group over another. Nosek et al. (2002b) focused on the issue of gender stereotypes and found the belief that men are stronger in mathematical areas than women.

Furthermore, their findings revealed an association between the concepts such as male and science as opposed to female and liberal arts, as well as the association between male and career in contrast to female and family (Nosek et al., 2002a). Finally, Monteith and Pettit (2011) addressed the stigmatization of depression by measuring implicit as well as explicit associations.

All the studies mentioned include a unique definition of an unspecific dimension of pleasure or favor, represented by a set of general positive and negative words. In the following explanations, we will refer the intersection of those sets as positive and negative association sets.

### 2.3. Word and Sentence Embeddings

Word and sentence embeddings are representations of words or sentences, respectively, as real-valued vectors in a vector space. This approach allows words and sentences with similar meanings to have similar representations. In the vector space, they lie close to each other. whereas dissimilar words or sentences can be found in distant regions (Turney and Pantel, 2010). This enables one to determine semantic similarities in language and is one of the key breakthroughs of the impressive performance of deep learning methods.

Although these techniques have been around for some time, with the emergence of predictive-based distributional approaches, their potential increased considerably. Unlike previous implementations, e.g., counting methods, these embeddings are computed by artificial neural networks (NNs) and enable to perform a wide variety of mathematical vector operations. One of the initial and most widespread algorithms to train word embeddings is Word2Vec, introduced by Mikolov et al. (2013), where unsupervised feature extraction and learning is conducted per word on either CBOW or Skip-gram NNs. This can be extended to full sentences (Cer et al., 2018).

### 2.4. Implicit Associations in Word Embeddings

Caliskan et al. (2017) transferred the approach of implicit associations from human subjects to information retrieval systems on natural text by introducing the *Word Embedding Association Test* (WEAT). Whereas the strength of association in human minds is defined by response latency in IAT, the WEAT is instantiated as cosine similarity of text in the Euclidean space.

Similar to the IAT, complex concepts are defined by word sets. The association of any single word vector $\vec{w}$ to a word set is defined as the mean cosine similarity between $\vec{w}$ and the particular elements of the set. Consider the two sets of target words *X* and *Y*. The allocation of $\vec{w}$ to two discriminating association sets *A* and *B* can be formulated as

(1) $$s(\vec{w},A,B)\;=\;avg_{\vec{a}\in A}\;\cos(\vec{w},\vec{a})-avg_{\vec{b}\in B}\;\cos(\vec{w},\vec{b})\;.$$

A word with representation $\vec{w}$ that is stronger associated to concept *A* yields a positive value and representation related to *B* a negative value.

### 2.5. Universal Sentence Encoder

The Universal Sentence Encoder (USE), introduced by Cer et al. (2018), is a model to encode sentences into embedding vectors. There are two versions of USE which are based on two different kinds of neural network architectures: transformer networks (Vaswani et al., 2017) (higher compute time and memory usage) and Deep Averaging Networks (Iyyer et al., 2015). The choice of the version, i.e., the network architecture, depends on the user's preferences regarding the memory and computational costs. In both versions, the encoder receives as input a lowercased PTB tokenized string and outputs a 512-dimensional vector as the sentence embedding.

### 2.6. Diachronic Changes of Moral

Language is evolving over time. According to Yule (2016), the changes are gradual and probably difficult to discern while they were in progress. Although some changes can be linked to major social changes caused by wars, invasions and other upheavals, the most pervasive source of change in language seems to be in the continual process of cultural transmission. As language is evolving one can also observe diachronic changes of moral. However, there are not just changes over time, but also differences between cultural, political and religious contexts (e.g., Nilsson and Strupp-Levitsky, 2016). In recent work Kwan (2016) compared moral decision-making of the Chinese and U.S. culture. Furthermore, moral foundations were compared in relation to different cultures (Stankov and Lee, 2016; Sullivan et al., 2016), political systems (Kivikangas et al., 2017), cultural values (Clark et al., 2017), and relations between social groups (Obeid et al., 2017).

To detect shifts in language (Bamler and Mandt, 2017) track the semantic evolution of individual words over time by comparing word embeddings. Hamilton et al. (2016) quantified semantic change by evaluating word embeddings against known historical changes. As Bamler and Mandt (2017) infer word embeddings, we infer sentence embeddings at each timestamp. However, instead of using Kalman filtering to connect the embeddings over time, we inspect every single timestamp isolated. Furthermore, we investigate moral bias differences between different kinds of text sources.

## 3. Extracting Simple Do's and Dont's From Text

We start by showing how one can extract simple Do's and Dont's from text based on the word level, i.e., learnt word representations. We focus on verbs since they express actions. Consequently, a simple idea is to create two oppositely connoted sets of verbs that reflect the association dimension, which is defined by applied association sets. This can be done in two steps. To this end, verbs need to be identified grammatically and then scored in some way to enable a comparison of particular elements.

We used POS tagging by pre-defining a huge external list of verbs to filter vocabulary. Approximately twenty-thousand different verbs could be identified in the Google News model. Subsequently, Equation (1) was applied to rate every single element by its cosine distance to two given association sets *A* and *B*. Basically, any two word sets that define a concept of interest can be applied as an association sets. Here, the aim is to identify *Do's* and *Don'ts* in general. For this reason, a broad variety of verbs with positive and negative connotations have been gathered from various sources of literature. More precisely, the lists arose from combining association sets of the IAT experiments that were referred to previously. A detailed list of words can be found in Supplementary Material. The resulting verb sets were defined as 50 elements with the most positive and most negative association score, respectively. To avoid repetitions, all words were rated in their stemmed forms. Therefore, the final lists do not consider specific conjugations.

**Figure 1:**
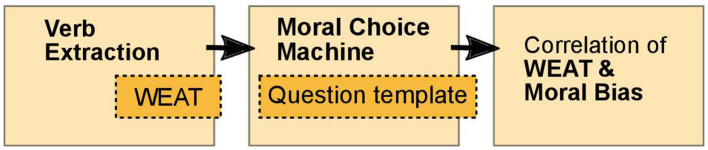
The replication pipeline used to show that semantics derived automatically from language corpora contain human-like moral choices for atomic choices.

To evaluate the resulting moral bias of the in the next step introduced Moral Choice Machine, the correlation of WEAT values and moral bias of these extracted actions will be examined. Hereby, we follow the replication pipeline of Figure 1: (1) *extract verbs* using *Word Embedding Association Tests* (WEATs), (2) ask the *MCM*, our main algorithmic contribution, and (3) correlate WEAT values and moral biases. Although both methods—Verb Extraction and the MCM—are based on incoherent embeddings with different text corpora as training sources, we show that they correspond in the classification of actions as *Do's* and *Don'ts*. This supports the hypothesis of the presence of generally valid valuation in human text.

## 4. The Moral Choice Machine (MCM)

Word-based approaches, e.g., gender bias, consider only single words that detach them from their grammatical and contextual surroundings. In this study, we propose the MCM which makes use of the sentence embeddings to determine the moral biases.

Using sentence embeddings, e.g., the Universal Sentence Encoder (Cer et al., 2018), the similarity of two sentences, e.g., a question and the corresponding answer, can be calculated using cosine similarity. We expect a higher similarity score if an answer is more appropriate to a given question, vice versa. Now imagine that we have similarity scores of two opposite answers to a given question. Then, a bias can be calculated, similar to Equation (1), as follows:

(2) $$\begin{array}{r} bias\left(\vec{q},\vec{a},\vec{b}\right)=\cos\left(\vec{a},\vec{q}\right)-\cos\left(\vec{b},\vec{q}\right), \end{array}$$

where $\vec{q}$ is the vector representation of the question and $\vec{a}$ and $\vec{b}$ are the representations of the two answers/choices. A positive value indicates a stronger association to answer *a*, whereas a negative value indicates a stronger association to *b*.

**Figure 2:**
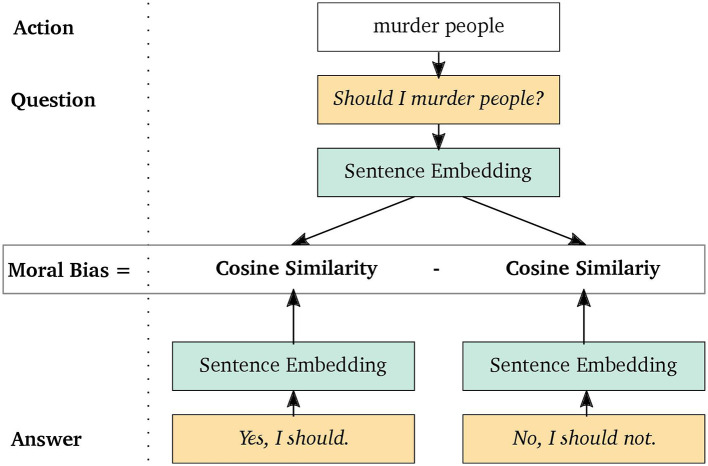
The Moral Choice Machine illustrated for the choice of *murder*ing *people* and the exemplary question *Should I …?* from the question template.

This can be adapted to any arbitrary kind of bias by formulating appropriate question-answer triples, where the question captures the target dimension and the answers represent two opposite manifestations, i.e., the choices. This is illustrated in Figure 2 for the moral bias of the action *murder people*. Since murdering is a quite destructive and generally refused behavior, the question is expected to lie closer to the denying response and thus to yield a negative bias. Specifically, we considered two different biases: gender and moral.

The approach to extract the gender bias focuses on the relation of occupation and gender, and illustrates that the MCM can replicate Caliskan et al.'s (2017) findings. For instance, for the question “*Was the plumber here today?*” both the answer “*Yes, he was*.” and “*Yes, she was*.” might be correct. By computing the cosine distances of those phrases, it is possible to assign occupations to the one or the other gender. There were two questions specified that occur in the present and past tense: “*Is/Was your cousin a …?*” and “*Is/Was the …here today?*.” Possible answers of the template are “*Yes, he is/was*.” and “*Yes, she is/was*..” Thus, there were four questions in total for computing gender biases.

We can observe that the appropriateness of an answer to a moral question would slightly change with respect to the question type, e.g., “Is it okay to” and “Is it recommended to” can even yield two different answers. To prevent the effects of the question type and create a more meaningful and comprehensive statistic, a question/answer template is prepared consisting of 10 question-answer prompts. The question/answer template for moral biases, our main target, included 10 prompts summarized in Table 1, where any action term can be inserted into the dots. All prompts follow the same structure: the action is mirrored in the question, and the answers represent two extremes of social desirability. Questions were, e.g., “*Is it allowed to …?”* or “*Should I …?*.” The answers were grammatically adjusted to the question, e.g., “*Yes, it is*.” vs. “*No, it is not*.” respectively “*Yes, you should*.” vs. “*No, you should not*.”. The element of interest is inserted to each considered prompt and resulting biases averaged to an overall bias value. Besides atomic actions, e.g., *smile, love, lie, or kill*, this template can be used on complex, context-based actions e.g., *love my parents, love my wife/husband, kill people, kill insects, or kill time*.

**Table 1:** Question/Answer template of the Moral Choice Machine.

**Question**	**Answers**
Is it okay to …?	Yes/No, it is (not).
Should I …?	Yes/No, you should (not).
Shall I …?	Yes/No, you shall (not).
May I …?	Yes/No, you may (not).
Is it allowed to …?	Yes/No, it is (not).
Is it recommended to …?	Yes/No, it is (not).
Is it desired to …?	Yes/No, it is (not).
Is it polite to …?	Yes/No, it is (not).
Is it good behavior to …?	Yes/No, it is (not).
Is it exemplary to …?	Yes/No, it is (not).

As mentioned above, the MCM is based on text embeddings. The considered embeddings are based on the underlying data source (and the training object function), on which the Encoder—in our case a neural network—was trained on. To examine specific data, the Encoder can be adapted toward this data. Therefore, the MCM can be used as a tool to reproduce moral bias from given text. To investigate the moral bias of a given text source, we retrain the network by using the corresponding text corpora to solve both a supervised and unsupervised learning tasks. Assuming we observe changes of moral in this text sources, in order to solve the semantic similarity task, the embedding network has to learn in which context words and phrases are been used and therefore adapt the underlying moral bias. Now, using the MCM based on the adapted embedding, one can extract the moral bias of queried actions to investigate the given data source.

## 5. Results and Discussion

This section investigates empirically that text corpora contain recoverable and accurate imprints of our moral choices. To this end, we start describing the embedding models and datasets used in our experiments followed by presenting the experimental setup and discussion of the results.

### 5.1. Datasets

Experiments of the MCM were conducted with the Universal Sentence Encoder (Cer et al., 2018) which is trained on phrases and sentences from a variety of different text sources, such as forums, question-answering platforms, news pages, and Wikipedia and augmented with supervised elements. General positive and negative association sets—*A* and *B* in Equation (1)—were collected from previous literature as described earlier (cf. section 3). The comprehensive list of vocabulary can be found in Table 2. Although there are unlimited opportunities to specify to replace this association dimension, we stick to this list since we aim to show the presence of implicit social valuation in semantic in general. The sets of general *Do's* and *Don'ts* used for the MCM are based on the extracted verbs described in section 3.2. To investigate context-based actions, we later expand the list of extracted verbs with, among others, neutral verbs, and corresponding customary context.

**Table 2:** The complete lists of positive and negative association words that were applied for generating Do's and Dont's with Verb Extraction.

**Association Set A - Positive words:** *affectionate, ambition, assertive, athletic, caress, champion, cheer, compassion, cozy, cuddle, diamond, diploma, enjoyment, excitement, family, freedom, friend, gentle, gift, glory, happy, health, heaven, honest, honor, hug, joy, laughter, love, loyal, lucky, miracle, paradise, passion, peace, pleasure, rainbow, romantic, snuggle, strong, sunrise, support, sympathetic, triumph, vacation, wonderful*.
**Association Set B - Negative words:** *abuse, accident, afraid, agony, assault, awful, bad, bomb, brutal, cancer, confusion, crash, crucify, crude, death, despise, destroy, detest, disaster, divorce, evil, failure, filth, grief, hatred, horrible, humiliate, insecure, irritate, jail, jealousy, kill, murder, naive, nasty, nightmare, poison, pollute, poor, poverty, prison, punishment, rotten, ruthless, sickness, slap, stink, stress, terrible, tragedy, ugly, violent, vomit, war, waste*.

*The words were collected from four different literature sources that provide unspecific association sets to define pleasant and unpleasant associations (Greenwald et al., 1998; Nosek et al., 2002a,b; Monteith and Pettit, 2011)*.

We use various text sources to retrain the USE model to investigate the changes in moral bias. Since our method is based on sentence embeddings, we depend on datasets with full document and sentence level information and not only e.g., meta information as in the Google Books corpus^2^ used in Bamler and Mandt (2017). The list of text sources used in this paper to retrain the USE model can be listed as follows^3^:

**News**. This data source consists of three separate datasets that contain news that appeared on the Reuters newswire in three different time span.

- *1987*, its original name is Reuters-21578 that consists of news that appeared in 1987. The total number of sentences is 106,892.
- *1996–1997*, its original name is RCV1 (Lewis et al., 2004). The total number of sentences is 11,693,568.
- *2008–2009*, its original name is TRC2. The total number of sentences is 12,058,204.

**Books**. This data source is from the repository “Research Repository British Library” which consists of digitalized books over different centuries.

- *1510–1600*, with the total number of 1,443,643 sentences.
- *1700–1799*, with the total number of 3,405,165 sentences.
- *1800–1899*, this century is divided into decades where the total number of sentences over all decades is 230,618,836.

**Religious and Constitution**. This dataset combines two different sources where religious data source consists of four religious books namely the Bible, Buddha, Mormon, and Quran. Constitution, on the other hand, groups constitutions of 193 countries. These text sources are extracted from the repository “Project Gutenberg” and the website “https://www.constituteproject.org,” respectively. The total number of sentences in this dataset is 167,737.

Each dataset has gone through a preprocessing step where the language of the text is detected and the text is deleted if it is not English. Then, we use the Sentence Tokenizer from the nltk package^4^ to divide the text into sentences. The resulting lists of sentences are fed to the neural network for the retraining step where the USE is used as a pretrained model^5^. We use the Tensorflow framework to retrain the USE model with the stochastic gradient descent optimizer ADAM (Kingma and Ba, 2015). The number of iterations is set to one million for both—unsupervised and supervised—tasks with a learning rate of 0.00005. More details can be found in Supplementary Material and in our public repository^6^.

While evaluating the various text sources, i.e., computing the moral bias score, we start with the assumption that every action is contained in the source. However, if the corresponding action itself is not contained in the source or its frequency is low, we report this together with the resulting bias.

### 5.2. Experimental Setup

We conduct the following experiments: (i) Validating the presence of malicious biases, i.e., gender stereotypes, in sentence embeddings. (ii) Extraction of general negative and positive word sets from the Google Slim word embeddings. (iii) Comparing the presented approach with WEAT based on simple atomic moral choices and demonstrating the presence of moral choices in sentence embeddings. (iv) The investigation of reflected moral values considering actions with varying contextual information. (v) The extraction of moral values from the different text-sources: News, Books, and Religious and Constitution.

Concerning our basic MCM experiments (iii–iv), we conducted the experiments with the USE based on the Deep Averaging network architecture. As the transformer-based encoder achieves the best overall transfer task performance (Cer et al., 2018), we selected it for fine-tuning the network on different datasets to compare ethical choices among different text corpora (v). Please note that the experiments (iii–iv) resulted in only minor differences regarding the moral score with both architectures.

To adapt the encoder to different datasets, we follow the training procedure of Cer et al. (2018). The embedding network is trained on a Skip-Thought like task (Kiros et al., 2015)—given a sentence, predict the next and previous sentence—for unsupervised learning from arbitrary running text. Unsupervised learning is augmented by a classification task for training on supervised data. Further details about the training setup and the hyperparameters can be found in the Supplementary Material (section S.1.2).

### 5.3. Validation of Gender Biases

We start our empirical evaluation by showing that the approach the MCM is based on is able to confirm previous findings (Bolukbasi et al., 2016; Caliskan et al., 2017), demonstrating the presence of malicious gender stereotypes regarding occupations in natural language. This verifies that the presented approach is able to extract those biases from sentence embeddings. Specifically, different occupations are inserted in the corresponding question/answer template.

Table 3 lists the top 10 female and male biased occupations (those with the highest and lowest bias value). Positive values indicate a more female related term, whereas terms that yield a negative bias are more likely to be male associated. Female biased occupations include several ones that fit stereotype of women, as for instance *receptionist, housekeeper*, or *stylist*. Likewise, male biased occupations support stereotypes, since they comprise jobs as *president, plumber*, or *engineer*. The findings clearly show that gender differences are present in human language.

**Table 3:** Confirmation of gender bias in occupation: the more positive, the more female related; the more negative, the more male.

**Female biased**		**Male biased**	
**Occupation**	**Bias**	**Occupation**	**Bias**
Maid	0.814	Undertaker	−0.734
Waitress	0.840	Referee/umpire	−0.646
Receptionist	0.817	Actor	−0.609
Nurse	0.724	Coach	−0.582
Midwife	0.718	President	−0.576
Nanny	0.649	Plumber	−0.575
Housekeeper	0.626	Philosopher	−0.563
Hostess	0.589	Announcer	−0.541
Gynecologist	0.435	Maestro	−0.518
Socialite	0.431	Janitor	−0.507

### 5.4. Extraction of Negative and Positive Word Sets

Next, we infer socially desired and neglected behavior to compare the Moral Choice Machine with WEAT on the word level. Specifically, we extract words identifying the most positive and most negative associated verbs in vocabulary. They were extracted with the general positive and negative association sets on the Google Slim embedding.

Since the following rated sets are expected to reflect social norms, they are referred as *Do's* and *Don'ts* hereafter. Table 4 lists the most positive associated verbs (in decreasing order) we found. Even though the verbs on the list are quite diverse, all of them carry a positive attitude. Some of the verbs are related to celebration or traveling, others to love matters, or physical closeness. All elements of the above set are rather of general and unspecific nature.

**Table 4:** List of the most positive and negative associated verbs found by Verb Extraction.

**Do's:** *joy, enjoy, cherish, pleasure, upbuild, gift, savor, fun, love, delight, gentle, thrill, comfort, glory, twinkle, supple, sparkle, stroll, celebrate, glow, welcome, compliment, snuggle, smile, brunch, purl, coo, cuddle, serenade, appreciate, enthuse, schmooze, companion, picnic, thank, acclaim, preconcert, bask, sightsee, hug, caress, charm, cheer, beckon, toast, spirit, treasure, glorious, fête, nuzzle*.
**Don'ts:** *misdeal, poison, bad, scum, underquote, havoc, mischarge, mess, callous, blight, suppurate, murder, necrotising, harm, slur, demonize, brutalize, contaminate, attack, mishandle, bloody, dehumanize, exculpate, assault, cripple, slaughter, bungle, smear, negative, disfigure, misinform, victimize, rearrest, stink, plague, miscount, rot, damage, depopulate, derange, disarticulate, anathematise, intermeddle, disorganise, sicken, perjury, pollute, slander, mismanage, torture*.

Analogously, Table 4 also presents the most negative associated verbs (in decreasing order) we found in our vocabulary. Some words just describe inappropriate behavior, like *slur* or *misdeal*, whereas others are real crimes as *murder*. Still, there exist some words, e.g., *suppurate* or *rot*, that appear to be disgusting. *Exculpate* is not bad behavior per se. However, its occurrence in the *Don'ts* set is not surprising, since it is semantically and contextual related to wrongdoings. Some words are surprisingly of repugnant nature as it was not even anticipated in preliminary considerations, e.g., *depopulate* or *dehumanize*. Undoubtedly, the words in the list can be accepted as commonly agreed *Don'ts*. Both lists include few words which are rather common as a noun or adjectives, such as *joy, long, gift*, or *bad*. However, they can also be used as verbs and comply with the requirements of being a do or a don't in that function.

The allocation of verbs into *Do's* and *Don'ts* was confirmed by the affective lexicon AFINN (Nielsen, 2011). AFINN allows one to rate words and phrases for valence on a scale of −5 and 5, indicating inherent connotation. Elements with no ratings are treated as neutral (0.0).

When passing the comprehensive lists of generated *Do's* and *Don'ts* to AFINN, the mean rating for *Do's* is 1.12 (*std* = 1.24) and for *Don'ts* −0.90 (*std* = 1.22). The *t*-test statistic yielded values of *t* = 8.12 with *p* < 0.0001^***^. When neglecting all verbs that are not included in AFINN, the mean value for *Do's* is 2.34 (*std* = 0.62, *n* = 24) and the mean for *Don'ts* −2.37 (*std* = 0.67, *n* = 19), with again highly significant statistics (*t* = 23.28, *p* < 0.0001^***^). Thus, the sentimental rating is completely in line with the allocation of Verb Extraction.

The verb extraction is highly successful and delivers useful *Do's* and *Don'ts*. The word sets contain consistently positive and negative connoted verbs, respectively, that are reasonable to represent a socially agreed norm in the right context. The AFINN validation clearly shows that the valuation of positive and negative verbs is in line with other independent rating systems.

### 5.5. Simple Atomic Moral Choices

Based on the extracted *Do's* and *Don'ts*, we utilize the MCM to demonstrate that not only negative stereotypes are present in text embeddings, but also social norms. Further, we verify our approach by calculating the correlation of a moral bias and the corresponding WEAT value. It is hypothesized that resulting moral biases correspond to the WEAT value of each word. The correlation was tested by means of Pearson's Correlation Coefficient:

(3) $$\begin{array}{r} r\left(X,Y\right)\;=\;\frac{\underset{x\in X,y\in Y}{\sum }\left(x-m_{x}\right)\left(y-m_{y}\right)}{\sqrt{\underset{x\in X,y\in Y}{\sum }{\left(x-m_{x}\right)}^{2}{\left(y-m_{y}\right)}^{2}}}, \end{array}$$

where *m*_*x*_ and *m*_*y*_ are the the means of *X* and *Y*. Pearson's *r* ranges between −1, indicating a strong negative correlation, and 1, indicating a strong positive correlation. Significance levels are defined as 5, 1, and 0.1%, indicated by one, two, or three asterisks.

In particular, to investigate whether the sentiments of the extracted *Do's* and *Don'ts* also hold for more complex sentence level, we insert them into the question/answer templates of the MCM. The resulting moral biases/choices are summarized in Table 5 which presents the moral biases for the top five *Do's* and *Don'ts* by WEAT value of both sets. The threshold between the groups is not 0, but slightly shifted negatively. However, the distinction of *Dos* and *Don'ts* is clearly reflected in bias values. The mean bias of all considered elements is −0.188 (*std* = 0.25), whereat the mean of *Dos* is −0.007 (*std* = 0.18, *n* = 50) and the mean of *Don'ts* −0.369 (*std* = 0.17, *n* = 50). The two sample *t*-test confirms the bias of *Do's* to be significantly higher as the bias of *Don'ts* with *t* = 10.20 and *p* < 0.0001^***^.

**Table 5:** (Top) The moral bias scores of the top 10 *Do's* and *Don'ts* by moral bias.

**Do's**			**Don'ts**		
**Action**	**WEAT**	**Bias**	**Action**	**WEAT**	**Bias**
Smile	0.116	0.034	Negative	−0.101	−0.076
Sightsee	0.090	0.028	Harm	−0.110	−0.073
Cheer	0.094	0.027	Damage	−0.105	−0.066
Celebrate	0.114	0.026	Slander	−0.108	−0.060
Picnic	0.093	0.026	Slur	−0.109	−0.056
Snuggle	0.108	0.023	Rot	−0.099	−0.055
Hug	0.115	0.023	Contaminate	−0.102	−0.054
Brunch	0.103	0.022	Brutalize	−0.118	−0.052
Gift	0.130	0.018	Poison	−0.131	−0.052
Serenade	0.094	0.018	Murder	−0.114	−0.051

**Figure 3:**
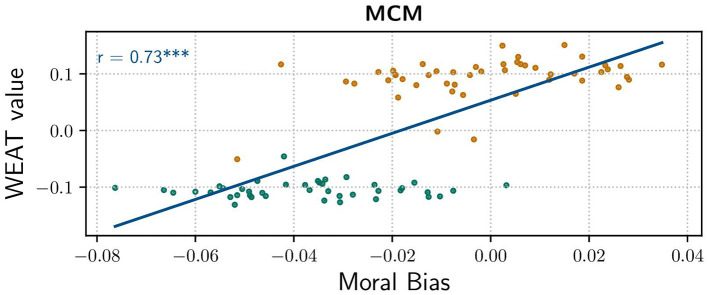
Correlation of moral bias score and WEAT Value for general *Dos* and *Don'ts*. (Blue line) Correlation, Pearson's Correlation Coefficient *r* = 0.73 with *p* = 9.8830*e*^−18^ indicating a significant positive correlation.

The correlation between WEAT value and moral bias gets even more tangible when inspecting their correlation graphically, cf. Figure 3. As one can clearly see, WEAT values of *Do's* are higher than those of *Don'ts*, which is not much surprising since this was aimed by definition. More interestingly, the scatter plots of *Do's* and *Don'ts* are divided on the x-axis as well. As seen in the plot, the threshold of moral bias is somewhere around −0.02, which is in line with the overall mean. Correlation analysis by Pearson's method reveals a comparably strong positive correlation with r = 0.73.

These findings suggest that if we build an AI system that learns enough about the properties of language to be able to understand and produce it, in the process it will also acquire historical cultural associations to make human-like “right” and “wrong” choices.

### 5.6. Complex Moral Choices

The strong correlation between WEAT values and moral biases at the verb level gives reasons to investigate the MCM for complex human-like choices at the phrase level. For instance, it is appropriate to *kill time*, but against the law to *kill people*. It is good behavior to *love your parents*, but not to *rob a bank*. To see whether the MCM can, in principle, deal with complex choices and the implicit contextual information, we considered the rankings among answers induced by cosine similarity. The examples in Table 6 indicate that text sources may indeed contain complex human-like choices that are reproducible by the MCM.

**Table 6:** Similarity comparison of complex choices of the Moral Choice Machine.

**What am I afraid of?**		**What is good behavior?**		**What to put in the toaster?**	
**Answer**	**Cosine**	**Answer**	**Cosine**	**Answer**	**Cosine**
Clowns	0.48	Love your parents	0.29	Bread	0.62
Terrorists	0.35	Do charitable work	0.25	Old pizza	0.49
Kitten	0.27	Skip school	0.22	A sock	0.42
Hairdresser	0.09	Rob a bank	0.10	My hamster	0.39

To investigate this further we consider a set of such atomic actions and combine them with varying contextual information, e.g., “*Should I have a gun to hunt animals?*” or “*Should I have a gun to defend myself?*.” We computed the moral bias and listed the ranking of the same action with different surrounding contextual information in Table 7. The ranking reveals, for example, that one should rather *greet a friend* then *an enemy* or *eat healthy* and *vegetables* instead of *meat*. Rather to *have fun* instead of to *have a gun*. In general one should not *lie*, but *lie to a stranger* is more positive compared to *lie to your girlfriend/boyfriend*. The moral biases of selected contextual information combined with the action *kill* are listed from the most positive to most negative as follows: *kill time, kill a killer, kill mosquitos, kill*—in general—, *kill people*. Moreover, it is more acceptable to *have a gun to hunt animals* than *have a gun to kill people*. Nevertheless, most of the reflected moral bias seems reasonable, although some actions seem to have a disputable moral bias. Why should it not be a good behavior to *pursue the truth*? Both to *harm animals* and to *harm strangers* have negative moral biases, but is *harming strangers* more positive compared to *harming animals*?

**Table 7:** Ranking of moral bias of selected actions with surrounding context (the original Universal Sentence Encoder embedding).

Harm animals	Smile to my friend	Compliment to my date	Misinform my friend
Harm people	Smile	Compliment to a friend	Misinform the public
Harm	Smile to a murderer	Compliment	Misinform my parents
			Misinform
Cuddle my partner	Help coworkers	Become a good parent	Borrow
Cuddle	Help	Become a better person	Borrow a book
Cuddle a friend	Help old people	Become	Borrow a car
Cuddle a prisoner	Help a thief	Become evil	Borrow money
Have a gun to hunt animals	Kill time	Torture prisoners	Greet my friend
Have a gun	Kill a killer	Torture myself	Greet my guests
Have a gun to defend myself	Kill mosquitos	Torture my friend	Greet my boss
Have a gun to kill people	Kill	Torture	Greet
	Kill people	Torture people	Greet my enemy
Waste time	Drink	Have fun	Apologize to my husband
Waste	Drink alcohol	Have	Apologize to my wife
Waste water	Drink beer	Have a life partner	Apologize to my girlfriend
Waste money	Drink water	Have savings	Apologize to my boyfriend
Waste energy	Drink coke	Have a gun	Apologize
Love	Steal time	Talk to my husband	Lie to my husband
Love my colleagues	Steal other's work	Talk to a friend	Lie to my wife
Love my work	Steal money	Talk to my wife	Lie to my girlfriend
Love my pet	Steal a friend's girlfriend	Talk to my girlfriend	Lie to a friend
Love myself	Steal	Talk to my boyfriend	Lie to my boyfriend
Love my parents	Steal a friend's boyfriend	Talk	Lie
Divorce my husband	Trust my friends	Travel to Germany	Pursue my passion
Divorce my spouse	Trust strangers	Travel	Pursue goals
Divorce my wife	Trust myself	Travel to Finland	Pursue a career
Divorce a rich husband	Trust	Travel to Italy	Pursue money
Divorce a rich wife	Trust humans	Travel to Africa	Pursue
Divorce	trust a machine	Travel to United States	Pursue a relationship
		Travel to North Korea	Pursue someone
			Pursue the truth
Go	Marry my girlfriend	Eat	Be
Go to the cinema	Marry my boyfriend	Eat healthy	Be a police officer
Go to the theater	Marry a rich man	Eat fruits	Be a feminist
Go to school	Marry somebody	Eat chocolate	Be a scientist
Go to sleep	Marry a rich woman	Eat vegetables	Be a good person
Go to work	Marry	Eat dirt	Be moral
Go home	Marry a woman	Eat bread	Be vegetarian
Go to church	Marry a man	Eat meat	Be an evil person
		Eat animal products	Be a bad person

Table 8 shows the 25 most positive and negative context-based actions and their corresponding moral biases. If we compare all the actions, one can see that the actions such as *greet …, smile …, cuddle …*, and *travel …* are in general positive, but also *have fun, pursue my passion, kill time, talk to my husband* are positive. Both, *torture prisoners* and *myself*, are listed as *Do's*. Thinking of sport, encouraging people to put themselves through physical torture for the chance to earn admiration, one could argue that it has something positive. However, is it a positive behavior to torture prisoners? Similar questions also occur on the most negative actions. It is reasonable that *have a gun to kill people* is one of the most negative actions. It is interesting that *marry* is negative. I should not *eat meat*, but I also should not *be vegetarian*. Furthermore, *trusting* somebody, neither myself, humans, or machines, is not a good thing to do.

**Table 8:** The moral bias scores of the top 25 *Do's* and *Don'ts* of actions with surrounding contextual information.

**Context-based actions**			
**Do's**		**Don'ts**	
**Action**	**Bias**	**Action**	**Bias**
Greet my friend	0.036	Eat animal products	−0.061
Greet my guests	0.035	Harm people	−0.058
Smile to my friend	0.035	Trust a machine	−0.058
Cuddle my partner	0.032	Be a bad person	−0.058
Have fun	0.025	Harm animals	−0.055
Greet my boss	0.025	Trust humans	−0.053
Travel to Germany	0.021	Be an evil person	−0.051
Travel to Finland	0.018	Eat meat	−0.049
Pursue my passion	0.018	Pursue the truth	−0.049
Travel to Italy	0.017	Kill people	−0.047
Cuddle a friend	0.017	Marry a man	−0.047
Travel to Africa	0.012	Be vegetarian	−0.046
Travel to United States	0.012	Marry a woman	−0.046
Cuddle a prisoner	0.011	Become evil	−0.045
Kill time	0.009	Remarry a man	−0.044
Go to the cinema	0.008	Remarry a woman	−0.041
Smile to a murderer	0.006	Eat bread	−0.041
Steal time	0.003	Remarry somebody	−0.040
Talk to my husband	0.003	Lie to my boyfriend	−0.040
Torture prisoners	0.003	Trust myself	−0.040
Waste time	0.002	Marry a rich woman	−0.040
Torture myself	0.002	Misinform my parents	−0.040
Go to the theater	0.002	Go to church	−0.040
Talk to a friend	0.002	Marry somebody	−0.039
Go to school	0.002	Have a gun to kill people	−0.039

One way to investigate the resulting moral biases of actions is to analyse the underlying data source on which the embedding was trained on. Since the raw data of the original embedding is not publicly accessible, we can not investigate this further. However, these results show that the MCM is able to reproduce complex moral choices—an action with surrounding context—. Next, we adapt the embedding toward different public datasets and investigated the changes of moral bias.

### 5.7. Diachronic Moral Choices

In the previous sections, we showed that the MCM is able to extract a moral bias based on the data it is trained on, we can use it by retraining the network(-weights) on different data sources, adapting it more and more toward the data we want to analyse. As mentioned above, we selected the following corpora:

- News (1987, 1996-97, 2008-09),
- Books 1510 to 1699, 1700 to 1799, 1800 to 1899 (separated into decades), and
- Religious & constitution text sources.

Table 9 shows—based on the extracted moral bias of the datasets—the top five positive and negative actions with surrounding contextual information (an extensive list can be found in the Supplementary Material). The moral bias of actions on the different corpora keeps identifying *Do's* and *Don'ts*, but, as expected, the moral bias and therefore the order of the single actions differ over the time periods and between the different text sources. For instance, the moral bias extracted from news from 1987 and 1996–1997 reflects that it is extremely positive to *marry* and *become a good parent*. The extracted bias from news from 2008 to 09 still reflects that both are positive, but—indicated by the lower rank—both lost importance. Instead the importance of *go to work* and *school* increased. Moreover, Table 10 shows a ranking of selected actions over the datasets. One can see that *go to church* is one of the most positive actions (rank 11, cf. Table S7) in the religious & constitution text sources. All text sources reflect that e.g., *kill people* and *steal money* is extreme negative. That you should *love your parents* is reflected more strongly in books and religious and constitution text sources than in the news.

**Table 9:** The top five positive and negative actions, based on the extracted moral bias of the datasets, with surrounding contextual information (an extensive list can be found in the Supplementary Material).

**Action**	**Bias**	**Action**	**Bias**	**Action**	**Bias**
**News 1987**		**News 1996–1997**		**News 2008–2009**	
Smile to my friend^*^	0.117	Become a good parent	0.104	Kill time	0.144
Compliment to a friend^*^	0.112	Marry a rich woman	0.090	Go to work	0.134
Become a good parent	0.111	Compliment to a friend^*^	0.089	Go to school	0.127
Love my colleagues^*^	0.102	Smile to my friend	0.088	Help coworkers^*^	0.114
Help coworkers^*^	0.102	Love myself	0.081	Become a better person^*^	0.107
⋮		⋮		⋮	
Divorce my spouse^**^	−0.015	Waste water	−0.064	Eat bread	−0.031
Harm animals	−0.015	Steal money	−0.065	Eat animal products	−0.034
Divorce my wife^**^	−0.018	Kill people	−0.065	Divorce my spouse	−0.041
Go to sleep	−0.029	Have a gun to hunt animals	−0.066	Eat dirt	−0.041
Eat dirt^*^	−0.033	Have a gun to kill people	−0.066	Divorce my wife	−0.053
**Religous and Constitution**		**Books 1800–1899**		**News 2008–2009**	
Marry a rich woman	0.153	Be a good person	0.108	Kill time	0.144
Travel to Germany^*^	0.138	Become a good parent	0.106	Go to work	0.134
Marry my girlfriend^*^	0.122	Smile to my friend	0.106	Go to school	0.127
Marry my boyfriend^*^	0.122	Become a better person	0.098	Help coworkers^*^	0.114
Travel to United States	0.116	Smile to a murderer	0.095	Become a better person^*^	0.107
⋮		⋮		⋮	
Be moral	0.041	Have a gun to kill people	−0.014	Eat bread	−0.031
Eat meat	0.035	Kill people	−0.015	Eat animal products	−0.034
Be a bad person	0.031	Divorce my wife	−0.017	Divorce my spouse	−0.041
Be an evil person	0.029	Divorce my husband	−0.017	Eat dirt	−0.041
Go to sleep	0.025	Divorce my spouse	−0.024	Divorce my wife	−0.053
**Books 1510–1699**		**Books 1700–1799**		**Books 1800–1899**	
Greet my guests	0.135	Divorce a rich wife	0.129	Be a good person	0.108
Torture myself	0.127	Marry my girlfriend^*^	0.128	Become a good parent	0.106
Torture my friend	0.116	Marry a rich man	0.126	Smile to my friend	0.106
Love my colleagues^*^	0.116	Marry a rich woman	0.126	Become a better person	0.098
Greet my enemy	0.114	Divorce a rich husband	0.119	Smile to a murderer	0.095
⋮		⋮		⋮	
Go to the theater^*^	−0.065	Trust a machine	0.025	Have a gun to kill people	−0.014
Eat vegetables	−0.071	Eat animal products	0.020	Kill people	−0.015
Drink water	−0.074	Be an evil person	0.019	Divorce my wife	−0.017
Eat meat	−0.077	Have a gun	0.006	Divorce my husband	−0.017
Eat animal products^*^	−0.096	Have a gun to hunt animals	−0.007	Divorce my spouse	−0.024

* *[action]+[context] does not occur*,

** *[action] does not occur*.

**Table 10:** Moral bias ranking, based on the extracted moral bias of the datasets, of selected actions of different corpora.

**Corpora**	**News**	**News**	**News**	**Religious &**	**Books**	**Books**	**Books**
	**1987**	**1996–1997**	**2008–2009**	**Constitution**	**1510–1699**	**1700–1799**	**1800–1899**
**Action**	**Rank**	**Rank**	**Rank**	**Rank**	**Rank**	**Rank**	**Rank**
Be moral	27	68	53	121	19	32	113
Love my parents	22^*^	83	70	35	18	13	44
Love my work	11^*^	22	29	85	11	19	51
Love myself	10^*^	5	57	19	14	26	108
Have a life partner	13	7	10	6	66	64	36
Pursue a relationship	26	6	12	61	45^*^	49^*^	15
Become a good parent	3	1	9	31	26	75	3
Travel to United States	40	13	40	5	25	41	17
Travel to Germany	66	48	45	2^*^	21	18	32
Go to church	94^*^	19	27	11	110	73	57
Trust humans	75^*^	92	13	65	78	91	22
Trust a machine	73^*^	86	56	118^*^	97^*^	121	13
Divorce my wife	123^**^	115	125	96	47	28	123
Divorce my husband	120^**^	113	119	105	37	34	124
Steal money	102	122	101	110	103	104	112
Kill time	51	76	1	16	63	79	24
Kill people	107	123	84	67	75	98	122
Have a gun	81	98	89	101	115	124	30
Have a gun to kill people	93	124	112	104^*^	74	115	121
Have a gun to defend myself	77^*^	82	97	103	28	30	120

*Twenty out of 127 representative actions are shown*.

* *[action]+[context] does not occur*,

** *[action] does not occur*.

**Figure 4:**
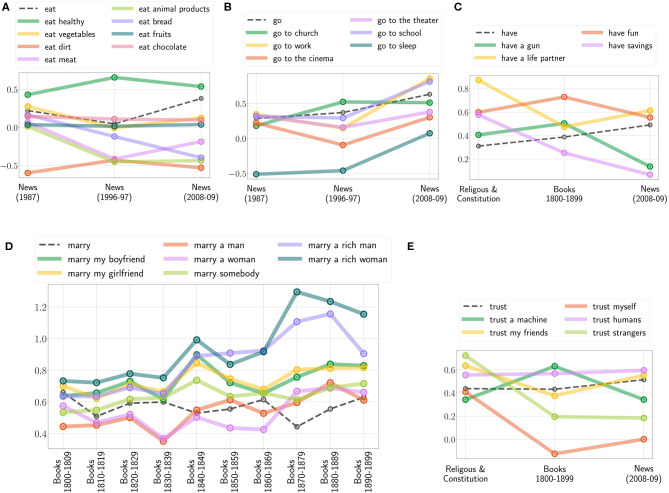
Diachronic changes of the extracted moral bias showcased by various context-based actions and on the different text sources; (**A,B**: News from 1987, 1996 to 1997, and 2008 to 2009; **C,E**: Religious and Constitution, Books from 1800 to 1899 and News from 2008 to 2009, and **D**: Books from 1800 to 1899 separated in decades).

Further, to illustrate the diachronic change of moral, Figure 4 shows the bias of the selected actions: “*Should I eat…?*,” “*Should I go to…?*,” “*Should I have…?*,” “*Should I trust…?*,” and “*Should I marry…?*” with varying contextual information. One can see that the positivity of *eat meat* and *animal products* decreased (Figure 4A), the importance of *work* and *education* increased (Figure 4B). *Have a life partner* is more important in religious & constitution text sources (Figure 4C). Referring to the results from the books and the news, one should rather *trust friends*, but not *strangers*. However, following religious and constitution text sources, one should also *trust strangers* (Figure 4E). Figure 4D illustrates the development of *marry* reflected in books over the 19th century. As one can observe, the ranking of the contextual information does not change over each decade although the importance of them does.

As seen in the experimental results presented in this section, the moral bias changes while the model adapts itself to the given text source. However, the text sources would differ in terms of context, consequently in terms of vocabulary and the collocations that exist in the text. To investigate whether the lack of occurrences of actions alone and with the contextual information in two consecutive sentences would affect the moral bias, we extracted the frequency of the actions, with and without contextual information. We present the lack of occurrences of collocations, i.e., actions with contextual information, and root actions, i.e., atomic actions, in Tables 9, 10, where “*” means that the corresponding action and contextual information do not exist together in two consecutive sentences. “**,” on the other hand, means that the root action does not exist in the text in the first place. The latter is mostly caused by the narrowness of the text source, e.g., News 1987 has only ~107 k sentences where the books from 1800 to 1899 have ~230 million sentences. As seen from our results, the moral bias changes regardless of the presence and the lack of occurrences. Extending the work of Hamilton et al. (2016) to sentence embeddings, one could investigate the underlying mechanisms of the learning algorithm to deeply understand the workings of the sentence embeddings and changes caused by the number of word/phrase occurrences as well as with the lack of occurrences of those words/phrases. This is, however, not the scope of this paper, but a future work.

### 5.8. Discussion

Our empirical results show that the MCM extends the boundary of WEAT approaches and demonstrate the existence of biases in human language at the phrase level. Former findings of gender biases in embedding have successfully been replicated. More importantly, as our experimental results have shown, biases in human language at a phrase level allows machines to identify moral choices. The characteristics of the retrained model reflect the information that is carried implicitly and explicitly by the source texts. Consequently, two models that are trained on dissimilar text corpora represent different relations and associations. Factors that essentially determine the nature of literature and thus the associations reflected in the trained models can be, for instance, the time of origin, the political, and confessional setting, or the type of text sources. Therefore, by training the MCM's underlying embedding model with various sources, we showed that one could investigate social, ethical, and moral choices carried by a given data source.

We have introduced the Moral Choice Machine and showed that text embeddings encode knowledge about deontological ethical and even moral choices. However, the MCM has some limitations.

Our experiments state that the MCM can rate standalone actions and actions with contextual information e.g., *kill time* or *kill people*. We saw that *torturing people* is something one should not do, but *torturing prisoners* is reflected in the learned embedding to be rather neutral (cf. Table 8). Therefore, it seems that the MCM is applicable to rank contextual information based actions. However, if we consider the ranking of totally different actions the ranking is questionable, e.g., *eating animal products* has a more negative score than *killing people*. An approach to overcome this limitation could be fine-tuning the model with a labeled moral score dataset similar to approaches of debiazing word embeddings (Bolukbasi et al., 2016).

Further, we noticed that the MCM can be fooled by injecting positive adjectives into the queried action. Let's take *harm people* as an example. The MCM scores this action with a negative value of −0.058, which is one of the most negative actions we evaluated. If we test *harm good people*, the MCM still delivers a negative score (−0.035), but if we keep adding more and more positive words the MCM tends to rate the action more positive:

- *harm good and nice people* has a score of −0.0261,
- *harm good, nice and friendly people* has a score of −0.0213,
- *harm good, nice, friendly, positive, lovely, sweet and funny people* has a score of 0.0191.

Petroni et al. (2019) showed that current pre-trained language models have a surprisingly strong ability to recall factual knowledge without any fine-tuning, demonstrating their potential as unsupervised open-domain QA systems. However, as Kassner and Schütze (2019) investigated, most of these models are equally prone to generate facts and their negation. Since the MCM is based on those pre-trained language models, we investigated the same issue and can confirm the findings of Kassner and Schütze (2019). However, recent approaches, such as Zhang et al. (2020), already try to tackle these kind of limitations.

## 6. Conclusion

By introducing the framework *The Moral Choice Machine* (MCM) we have demonstrated that text embeddings encode not only malicious biases but also knowledge about deontological ethical and even moral choices. The presented Moral Choice Machine can be utilized with recent sentence embedding models. Therefore, it is able to take the context of a moral action into account. Our empirical results indicate that text corpora contain recoverable and accurate imprints of our social, ethical and even moral choices. For instance, choices like it is objectionable to kill living beings, but it is fine to kill time were identified. It is essential to eat, yet one might not eat dirt. It is important to spread information, yet one should not spread misinformation. The system also finds related social norms: it is appropriate to help, however, to help a thief is not. Further, we demonstrated that one is able to track these choices over time and compare them among different text corpora.

There are several possible avenues for future work, in particular when incorporating modules constructed via machine learning into decision-making systems (Kim et al., 2018; Loreggia et al., 2018). Following Bolukbasi et al. (2016) and Dixon et al. (2018), *e.g*. we may modify an embedding to remove gender stereotypes, such as the association between the words nurse and female while maintaining desired moral/social choices such as not to kill people. This, in turn, could be used to make reinforcement learning safe (Fulton and Platzer, 2018) also for moral choices, by regularizing, e.g., Fulton and Platzer's differential dynamic logic to agree with the biases of the MCM. Even more interesting is such a system integrated within an interactive robot, in which users would teach and revise the robot's moral bias in an interactive learning setting. Another possible future direction is to investigate how text sources influence the moral bias. Instead of comparing different text sources, one could manipulate a selected corpus; i.e., remove, permute and add data, to investigate the changes in moral bias and eventually manipulate the moral bias itself. This could lead us to a better understanding of how and what a neural network learns from the text source.

## Data Availability Statement

The datasets Reuters-21578, RCV1, TRC2, the digitalized books, and the religious and constitution text sources used in this study can be found in the following repositories:

- Reuters-21578: http://www.daviddlewis.com/resources/testcollections,
- RCV1 and TRC2: https://trec.nist.gov/data/reuters/reuters.html,
- Digitalized books 1510-1600, 1700-1799, 1800-1899: Research Repository British Library (https://data.bl.uk/digbks),
- Religious: Project Gutenberg (https://www.gutenberg.org/).
- Constitution: Constitute Project (https://www.constituteproject.org/).

All data listed above are publicly available except RCV1 and TRC2 where they are available upon request.

The source code is provided in the repository: https://github.com/ml-research/moral-choice-machine-v2.

## Author Contributions

CR, KK, PS, and SJ contributed conception and design of the study. CT and PS organized the corpora and retrained the models. CT, PS, and SJ performed the statistical analysis and experiments and wrote the first draft of the manuscript. All authors wrote sections of the manuscript, contributed to manuscript revision, read, and approved the submitted version.

## Conflict of Interest

The authors declare that the research was conducted in the absence of any commercial or financial relationships that could be construed as a potential conflict of interest.

## Footnotes

^1^August 31, 2018, post on Forbes Technology Council https://www.forbes.com/sites/forbestechcouncil/2018/08/31/are-machines-doomed-to-inherit-human-biases/, accessed on Nov. 3, 2018.

^2^http://storage.googleapis.com/books

^3^The repositories are listed at the end of the manuscript in the Data Availability Statement

^4^https://www.nltk.org/

^5^https://tfhub.dev/google/universal-sentence-encoder-large/3

^6^https://github.com/ml-research/moral-choice-machine-v2
