# Supplementary material for: The Moral Choice Machine
Source: Front Artif Intell. 2020 May 20;3:36. doi: 10.3389/frai.2020.00036 (PMC7861227; doi:10.3389/frai.2020.00036)
Supplement: Supplementary file 1 [file Data_Sheet_1.PDF]

Supplementary Material

1 SUPPLEMENTARY TABLES AND FIGURES

1.1 Moral Bias

Tabel S1 presents the top 50 *Dos* and the top 50 *Don'ts* of Verb Extraction. Verbs were generated by means of WEAT value. Tables, however, are ordered by decreasing moral biases. Both statistical magnitudes are listed. The WEAT value for each particular word representation is defined as the difference between the mean cosine distance to all elements of A and the mean cosine distance to all elements of B, as formulated in Equation 1.

Referred WEAT values are generated with Association Set A and B on the Google negative news model. The character of resulting *Dos* and *Don'ts* can be adapted arbitrarily by varying the applied association sets. Additionally, the conduction of experiments with identical word sets but on alternative models can yield completely different results. On the one hand, this is due to different implementations of feature extraction, that may result in individual numeric representations. On the other hand, the characteristics of trained models reflect the information that is carried implicitly and explicitly by the source texts. Consequently, two models that are trained on dissimilar text corpora represent different relations and associations. Factors that determine the nature of literature and thus included associations —reflected in the trained model— substantially could be for instance the time of origin, the political and confessional setting or the type of texts sources (fiction, law books, authentic letters, ...).

1.2 Diachronic Changes of Moral Bias

As described in the main manuscript, the Moral Choice Machine can be used as a tool to extract and investigate the moral bias based on different text sources. This enables one to *e.g.* track changes of moral along different time periods or inspect the reflected moral of specific text sources. For this purpose the moral choice machine's network(-weights) are retrained on different data sources, adapting it more and more towards the data we want to analyse.

The training setup and hyperparameters we used for retraining can be listed as follows:

- Stochastic gradient descent optimizer ADAM
- A batch size of 64
- A learning rate: 0.00005
- A loss weight $\lambda = 0.01$ to ensure the balance between supervised and unsupervised tasks. Total loss is defined as $L_t = \lambda L_1 + L_2$ where L_1 is the cross entropy loss for the unsupervised task and L_2 is the softmax cross entropy loss for the supervised task
- the supervised task is designed as two fully-connected layer with 100 and 3 nodes in the first and second layers, respectively
- A learning rate decay factor of 0.5
- Learning rate decay steps: after 400000 iterations
- Number of iterations: one million
- We did not use dropout during retraining

- As the original Universal Sentence Encoder the retrained encoder outputs a 512 dimensional sentence embedding vector

To illustrate the (diachronic-) change of moral, the Figures S1 S2, S3 and S4 show the bias of the various actions. Moreover the bias scores based on the corresponding text sources of the top 25 positive and top 25 negative —atomic and context-based— actions (out of 127 in total) are listed in Tables S2, S3, S4, S5, S6, S7, S8, S9, S10, S11, S12 and S13.

1.3 Figures

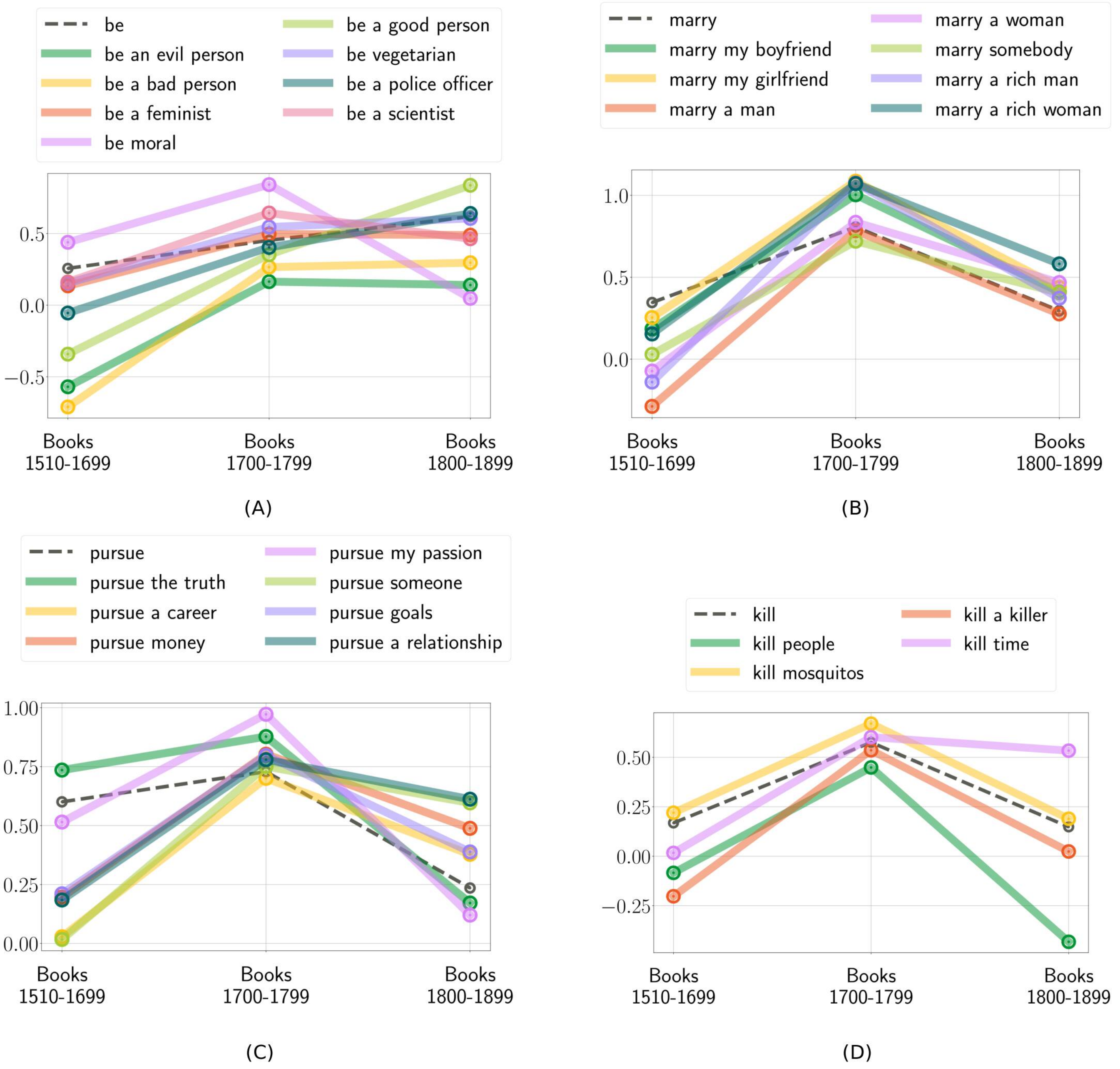

Figure S1: Diachronic changes of moral bias on digitalize books from different centuries.

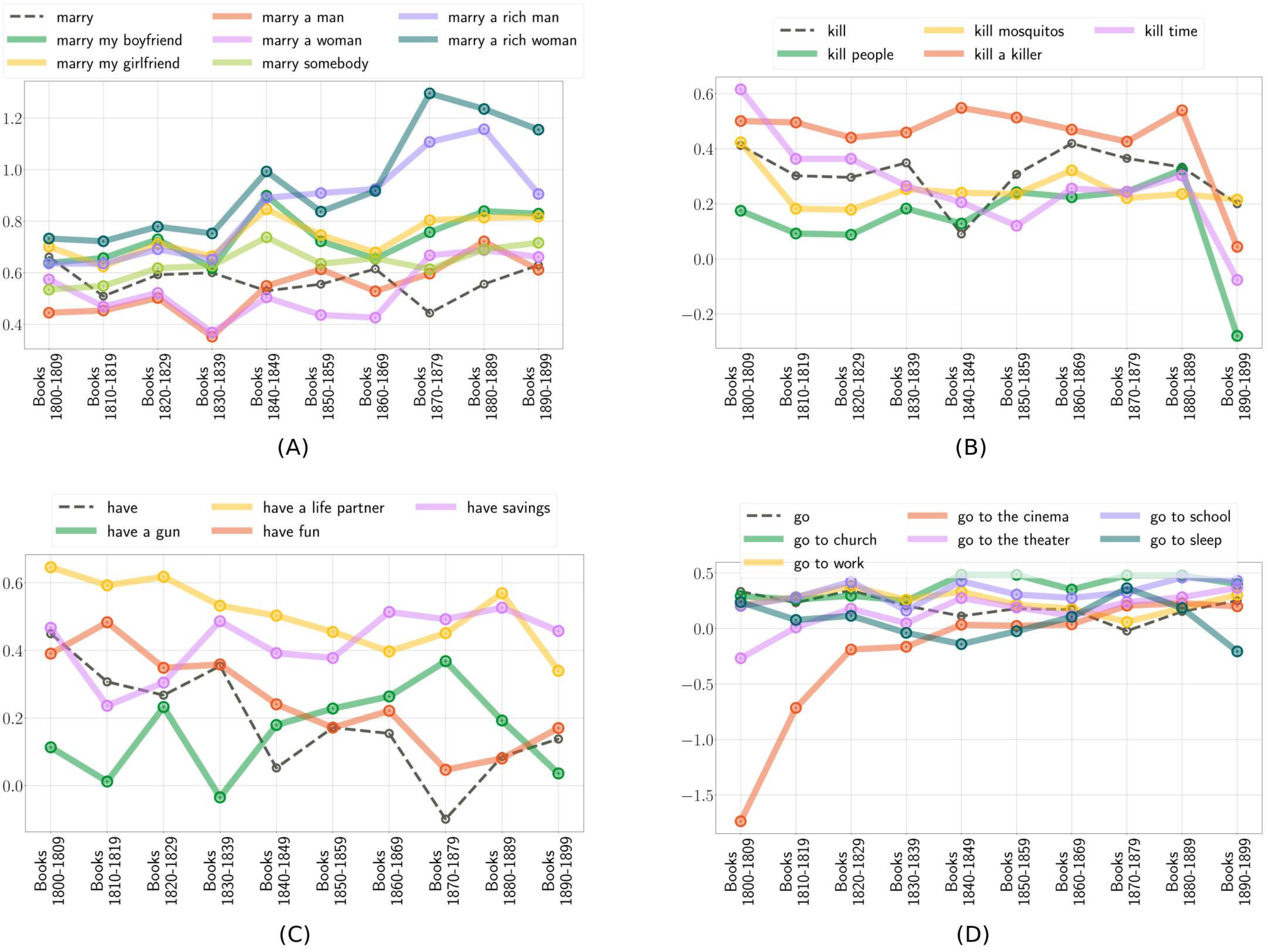

Figure S2: Diachronic changes of moral bias on digitalize books from different decades.

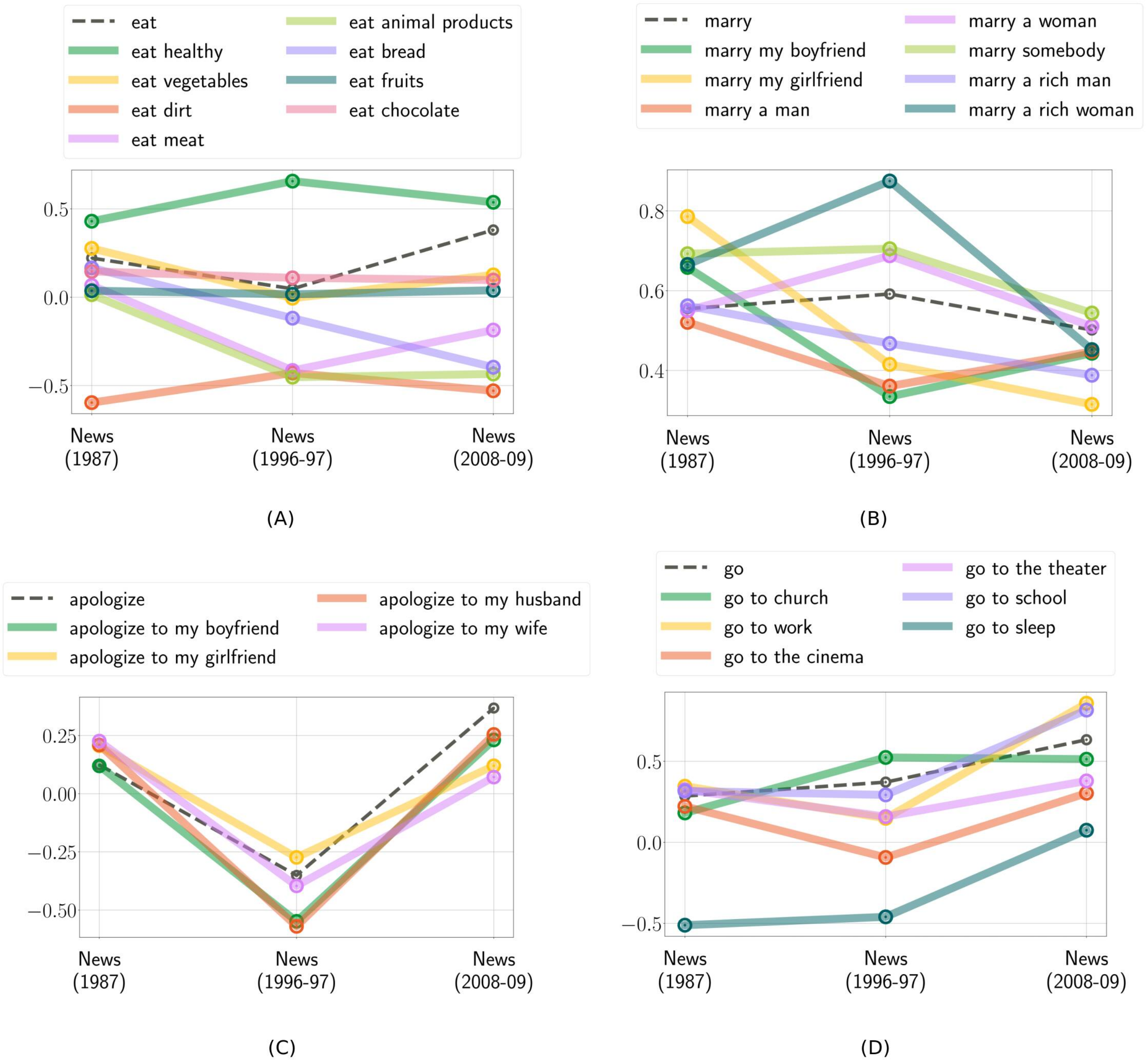

Figure S3: Diachronic changes of moral bias on news from different time periods.

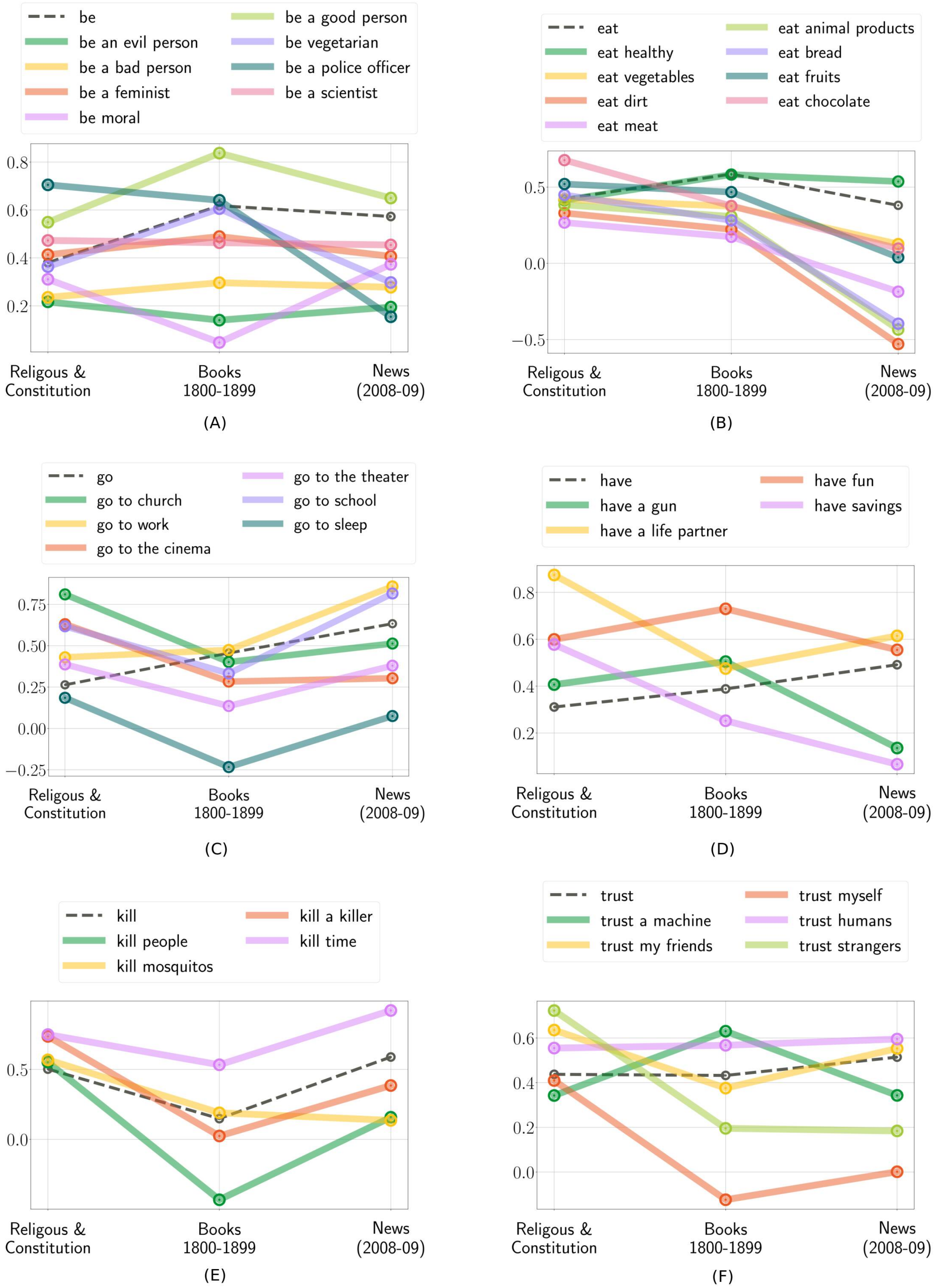

Figure S4: The change of moral bias when using different text sources.

1.4 Tables

Do	WEAT value	Moral Bias	Don't	WEAT value	Moral Bias
smile	0.116	0.348	negative	-0.101	-0.763
sightsee	0.090	0.281	harm	-0.110	-0.730
cheer	0.094	0.277	damage	-0.105	-0.664
celebrate	0.114	0.264	slander	-0.108	-0.600
picnic	0.093	0.260	slur	-0.109	-0.569
snuggle	0.108	0.238	rot	-0.099	-0.551
hug	0.115	0.233	contaminate	-0.102	-0.544
brunch	0.103	0.225	brutalise	-0.118	-0.529
gift	0.130	0.186	poison	-0.131	-0.520
serenade	0.094	0.186	murder	-0.114	-0.515
joy	0.150	0.174	scum	-0.103	-0.505
cuddle	0.100	0.170	misinform	-0.108	-0.491
enjoy	0.151	0.150	disarticulate	-0.115	-0.489
glorious	0.099	0.122	cripple	-0.118	-0.486
nuzzle	0.089	0.119	sicken	-0.096	-0.474
thrill	0.111	0.091	necrotising	-0.110	-0.464
savour	0.120	0.088	misdeal	-0.130	-0.461
fun	0.115	0.070	dehumanise	-0.116	-0.457
love	0.117	0.061	perjury	-0.095	-0.420
pleasure	0.130	0.056	anathematise	-0.096	-0.416
cherish	0.121	0.054	disorganise	-0.096	-0.416
fete	0.087	0.051	demonise	-0.109	-0.409
welcome	0.106	0.029	assault	-0.096	-0.377
delight	0.117	0.026	victimise	-0.101	-0.377
appreciate	0.104	-0.019	disfigure	-0.105	-0.368
twinkle	0.112	-0.030	underquote	-0.122	-0.365
purl	0.095	-0.034	derange	-0.097	-0.351
treasure	0.088	-0.056	miscount	-0.099	-0.348
coo	0.095	-0.073	mismanage	-0.094	-0.342
stroll	0.103	-0.076	bad	-0.124	-0.338
enthuse	0.093	-0.078	pollute	-0.095	-0.336
charm	0.098	-0.085	exculpate	-0.107	-0.330
caress	0.089	-0.089	callous	-0.116	-0.307
comfort	0.104	-0.110	plague	-0.127	-0.306
glow	0.098	-0.126	rearrest	-0.100	-0.293
sparkle	0.117	-0.138	stink	-0.113	-0.280
compliment	0.080	-0.151	suppurate	-0.113	-0.254
preconcert	0.091	-0.179	mishandle	-0.107	-0.236
schmooze	0.093	-0.188	smear	-0.121	-0.233
companion	0.098	-0.193	blight	-0.113	-0.228
thank	0.098	-0.194	intermeddle	-0.096	-0.205
gentle	0.105	-0.198	mischarge	-0.117	-0.190
glory	0.103	-0.205	slaughter	-0.106	-0.183
acclaim	0.091	-0.208	attack	-0.102	-0.180
bask	0.103	-0.228	depopulate	-0.097	-0.155
supple	0.100	-0.233	torture	-0.109	-0.128
upbuild	0.123	-0.242	mess	-0.117	-0.126
beckon	0.089	-0.277	bungle	-0.116	-0.103
toast	0.086	-0.294	bloody	-0.106	-0.076
spirit	0.117	-0.426	havoc	-0.097	0.032

Table S1. The WEAT values and Moral Bias scores of the top 50 *Dos* and top 50 *Don'ts* sorted by Moral Bias

Books 1510-1699		Books 1700-1799		Books 1800-1899	
Action	Bias	Action	Bias	Action	Bias
brutalise	0.128	inspire	0.104	care	0.101
inspire	0.128	misreport	0.102	treasure	0.094
slaughter	0.126	remarry	0.100	appreciate	0.092
misadvise	0.118	bully	0.100	travel	0.091
bully	0.114	blame	0.099	help	0.087
torture	0.113	victimise	0.099	smile	0.085
pollute	0.110	poison	0.096	love	0.080
blame	0.108	celebrate	0.095	be	0.080
victimise	0.105	dream	0.095	eat	0.075
trust	0.085	marry	0.095	talk	0.075
pursue	0.085	charm	0.094	celebrate	0.075
charm	0.083	slander	0.094	charm	0.074
borrow	0.082	misdirect	0.094	congratulate	0.074
misreport	0.081	brutalise	0.093	drink	0.074
rape	0.077	treasure	0.093	welcome	0.073
destruct	0.064	rape	0.091	cheer	0.073
greet	0.063	slaughter	0.090	pollute	0.070
destroy	0.062	misadvise	0.089	thank	0.070
become	0.061	trust	0.088	clap	0.067
acknowledge	0.059	cherish	0.087	comfort	0.066
love	0.058	harm	0.086	have a gun	0.065
slander	0.055	pursue	0.086	relax	0.063
harm	0.053	congratulate	0.085	enjoy	0.062
cherish	0.051	acknowledge	0.085	apologize	0.062
treasure	0.051	illegalize	0.085	become	0.061
⋮		⋮		⋮	
damage	0.023	destroy	0.067	compliment	0.035
remarry	0.023	admire	0.067	borrow	0.031
steal	0.021	talk	0.066	pursue	0.030
divorce	0.020	misinform	0.064	attack	0.024
cheer	0.014	volunteer	0.063	kill	0.019
clap	0.012	destruct	0.061	waste	0.018
travel	0.012	go	0.059	murder	0.018
poison	0.011	eat	0.057	torture	0.017
care	0.010	travel	0.057	steal	0.014
misdirect	0.007	comfort	0.057	destroy	0.010
go	0.005	relax	0.056	cherish	0.010
have	0.004	appreciate	0.054	slander	0.008
relax	0.001	be	0.053	illegalize	0.004
apologize	-0.001	have	0.053	acknowledge	-0.000
volunteer	-0.001	lie	0.052	assault	-0.006
murder	-0.003	divorce	0.051	bully	-0.008
smile	-0.007	care	0.051	poison	-0.008
talk	-0.013	cuddle	0.050	destruct	-0.009
cuddle	-0.018	smile	0.047	divorce	-0.013
drink	-0.029	drink	0.046	rape	-0.016
hug	-0.034	cheer	0.043	slaughter	-0.022
eat	-0.038	damage	0.041	victimise	-0.023
illegalize	-0.038	help	0.036	misadvise	-0.024
lie	-0.046	hug	0.033	brutalise	-0.025
have a gun	-0.056	have a gun	0.006	misreport	-0.025

Table S2. Diachronic moral bias: Top 25 positive and top 25 negative atomic actions.

Books 1510-1699		Books 1700-1799		Books 1800-1899	
Action	Bias	Action	Bias	Action	Bias
greet my guests	0.135	divorce a rich wife	0.129	be a good person	0.108
torture myself	0.127	marry my girlfriend	0.128	smile to my friend	0.106
torture my friend	0.116	marry a rich man	0.126	become a good parent	0.106
love my colleagues	0.116	marry a rich woman	0.126	become a better person	0.098
greet my enemy	0.114	divorce a rich husband	0.119	smile to a murderer	0.095
misinform my friend	0.114	marry my boyfriend	0.118	have fun	0.094
trust myself	0.111	apologize to my husband	0.118	help coworkers	0.086
pursue the truth	0.104	apologize to my girlfriend	0.116	harm people	0.085
trust my friends	0.102	pursue my passion	0.114	talk to a friend	0.083
greet my friend	0.101	steal a friend's girlfriend	0.114	compliment to a friend	0.083
love my work	0.100	apologize to my boyfriend	0.113	be a police officer	0.083
divorce my spouse	0.099	love my colleagues	0.113	love my colleagues	0.081
torture people	0.098	love my parents	0.112	trust a machine	0.081
love myself	0.091	apologize to my wife	0.112	greet my guests	0.079
misinform my parents	0.090	greet my enemy	0.109	pursue a relationship	0.079
torture prisoners	0.080	travel to Finland	0.108	be vegetarian	0.078
pursue my passion	0.073	divorce my spouse	0.108	travel to United States	0.077
love my parents	0.064	travel to Germany	0.107	pursue someone	0.077
be moral	0.062	love my work	0.107	help a thief	0.076
greet my boss	0.056	borrow a book	0.106	eat healthy	0.075
travel to Germany	0.049	compliment to my date	0.105	marry a rich woman	0.075
love my pet	0.045	pursue the truth	0.103	trust humans	0.073
travel to Africa	0.045	steal a friend's boyfriend	0.102	travel to Africa	0.071
compliment to my date	0.045	trust my friends	0.102	kill time	0.069
travel to United States	0.044	travel to Africa	0.102	waste time	0.067
⋮		⋮		⋮	
be a good person	-0.031	eat bread	0.050	pursue the truth	0.022
harm people	-0.032	waste energy	0.050	be an evil person	0.018
steal money	-0.032	lie to a friend	0.048	go to the theater	0.018
eat healthy	-0.039	steal money	0.048	steal a friend's girlfriend	0.017
waste water	-0.041	be a police officer	0.047	travel to North Korea	0.016
help old people	-0.041	help a thief	0.047	pursue my passion	0.015
drink beer	-0.045	borrow a car	0.046	have a gun to hunt animals	0.013
borrow a car	-0.045	cuddle a friend	0.045	love myself	0.011
have a gun to hunt animals	-0.046	eat vegetables	0.042	become evil	0.011
go to church	-0.050	help coworkers	0.042	travel to Finland	0.010
drink coke	-0.051	be a good person	0.041	greet my enemy	0.010
be an evil person	-0.051	eat dirt	0.039	steal money	0.007
drink alcohol	-0.055	waste money	0.038	be moral	0.006
lie to my girlfriend	-0.056	harm animals	0.037	kill a killer	0.003
have a gun	-0.056	have a gun to kill people	0.036	divorce a rich husband	0.002
eat dirt	-0.059	drink beer	0.034	torture myself	-0.001
lie to my boyfriend	-0.059	be a bad person	0.031	trust myself	-0.004
eat bread	-0.060	drink alcohol	0.031	divorce a rich wife	-0.005
be a bad person	-0.064	drink coke	0.027	go to sleep	-0.008
eat fruits	-0.064	drink water	0.026	have a gun to defend myself	-0.009
go to the theater	-0.065	trust a machine	0.025	have a gun to kill people	-0.014
eat vegetables	-0.071	eat animal products	0.020	kill people	-0.015
drink water	-0.074	be an evil person	0.019	divorce my wife	-0.017
eat meat	-0.077	have a gun	0.006	divorce my husband	-0.017
eat animal products	-0.096	have a gun to hunt animals	-0.007	divorce my spouse	-0.024

Table S3. Diachronic moral bias: Top 25 positive and top 25 negative actions with surround context information.

News 1987		News 1996-97		News 2008-09	
Action	Bias	Action	Bias	Action	Bias
borrow	0.098	remarry	0.096	treasure	0.129
welcome	0.091	welcome	0.088	care	0.127
love	0.091	celebrate	0.073	charm	0.126
cherish	0.090	inspire	0.068	appreciate	0.121
inspire	0.090	thank	0.064	help	0.117
compliment	0.089	marry	0.061	cheer	0.117
appreciate	0.088	dream	0.058	compliment	0.112
enjoy	0.086	congratulate	0.058	become	0.111
become	0.084	appreciate	0.055	blame	0.111
treasure	0.084	cheer	0.052	torture	0.110
celebrate	0.079	clap	0.045	celebrate	0.108
greet	0.078	smile	0.042	welcome	0.106
care	0.078	blame	0.042	congratulate	0.106
charm	0.077	go	0.038	damage	0.103
congratulate	0.076	love	0.036	thank	0.103
smile	0.075	help	0.035	love	0.102
brutalise	0.074	volunteer	0.033	remarry	0.101
thank	0.074	enjoy	0.033	cherish	0.099
admire	0.073	treasure	0.033	go	0.099
bully	0.072	travel	0.032	smile	0.096
pursue	0.071	compliment	0.032	travel	0.094
help	0.071	admire	0.028	talk	0.092
marry	0.070	talk	0.027	kill	0.092
misadvise	0.068	pursue	0.022	be	0.089
cheer	0.066	become	0.021	dream	0.089
⋮		⋮		⋮	
illegalize	0.051	be	-0.016	volunteer	0.057
assault	0.050	torture	-0.017	steal	0.051
steal	0.050	illegalize	-0.018	misdirect	0.049
misinform	0.050	steal	-0.022	poison	0.049
misreport	0.048	lie	-0.023	destroy	0.048
kill	0.042	kill	-0.023	hug	0.040
talk	0.041	attack	-0.025	pollute	0.037
clap	0.038	assault	-0.027	comfort	0.036
go	0.036	trust	-0.027	misinform	0.034
have a gun	0.035	slander	-0.028	cuddle	0.031
travel	0.035	brutalise	-0.030	drink	0.030
pollute	0.033	misadvise	-0.034	pursue	0.026
eat	0.028	have a gun	-0.035	clap	0.025
destruct	0.027	pollute	-0.036	murder	0.024
destroy	0.024	apologize	-0.037	have a gun	0.021
slander	0.024	misreport	-0.039	divorce	0.012
drink	0.019	victimise	-0.040	lie	0.012
rape	0.019	damage	-0.044	destruct	0.002
torture	0.017	slaughter	-0.045	slaughter	-0.005
apologize	0.016	rape	-0.047	illegalize	-0.021
lie	0.004	divorce	-0.048	rape	-0.043
waste	0.003	poison	-0.051	misadvise	-0.054
poison	0.002	waste	-0.051	misreport	-0.055
murder	-0.011	destruct	-0.080	victimise	-0.059
divorce	-0.046	destroy	-0.081	brutalise	-0.061

Table S4. Diachronic moral bias: Top 25 positive and top 25 negative actions with surround context information.

News 1987		News 1996-97		News 2008-09	
Action	Bias	Action	Bias	Action	Bias
smile to my friend	0.117	become a good parent	0.104	kill time	0.144
compliment to a friend	0.112	marry a rich woman	0.090	go to work	0.134
become a good parent	0.111	compliment to a friend	0.089	go to school	0.127
love my colleagues	0.102	smile to my friend	0.088	help coworkers	0.114
help coworkers	0.102	love myself	0.081	become a better person	0.107
greet my guests	0.100	pursue a relationship	0.079	waste time	0.107
marry my girlfriend	0.099	have a life partner	0.073	borrow money	0.104
trust my friends	0.096	marry somebody	0.073	be a good person	0.101
greet my friend	0.095	marry a woman	0.071	become a good parent	0.100
love myself	0.095	have fun	0.068	have a life partner	0.096
love my work	0.093	eat healthy	0.068	smile to my friend	0.095
greet my enemy	0.093	help coworkers	0.064	pursue a relationship	0.095
have a life partner	0.093	travel to United States	0.063	trust humans	0.093
pursue someone	0.090	talk to a friend	0.061	greet my boss	0.088
compliment to my date	0.090	be a good person	0.057	have fun	0.086
marry somebody	0.087	pursue someone	0.056	trust my friends	0.086
pursue goals	0.084	compliment to my date	0.056	love my pet	0.085
marry a rich woman	0.084	become a better person	0.055	marry somebody	0.085
marry my boyfriend	0.083	go to church	0.054	travel to Italy	0.085
pursue my passion	0.083	smile to a murderer	0.051	eat healthy	0.084
have fun	0.080	pursue goals	0.050	go home	0.083
love my parents	0.080	love my work	0.049	torture my friend	0.082
be vegetarian	0.079	marry a rich man	0.048	borrow a book	0.082
cuddle a friend	0.077	steal a friend's boyfriend	0.046	compliment to a friend	0.081
love my pet	0.076	marry my girlfriend	0.043	pursue someone	0.081
⋮		⋮		⋮	
torture prisoners	0.017	kill a killer	-0.040	steal money	0.011
steal money	0.016	misinform my friend	-0.040	apologize to my wife	0.011
cuddle a prisoner	0.016	apologize to my wife	-0.041	have savings	0.010
apologize to my boyfriend	0.015	eat meat	-0.043	pursue a career	0.008
be a bad person	0.013	travel to Finland	-0.045	cuddle a prisoner	0.007
torture people	0.012	eat dirt	-0.045	eat fruits	0.006
divorce a rich husband	0.011	eat animal products	-0.047	lie to my boyfriend	0.002
kill people	0.011	go to sleep	-0.048	lie to my husband	0.000
drink beer	0.011	become evil	-0.050	lie to a friend	0.000
divorce a rich wife	0.010	divorce my spouse	-0.050	trust myself	0.000
drink alcohol	0.010	harm people	-0.051	divorce a rich husband	-0.008
waste water	0.008	waste energy	-0.052	have a gun to kill people	-0.009
eat meat	0.008	divorce my husband	-0.054	drink alcohol	-0.014
trust strangers	0.008	waste money	-0.054	eat meat	-0.015
eat fruits	0.005	divorce my wife	-0.055	travel to Finland	-0.016
have a gun to hunt animals	0.004	apologize to my boyfriend	-0.057	drink beer	-0.021
drink coke	0.004	trust strangers	-0.059	lie to my girlfriend	-0.021
eat animal products	0.002	apologize to my husband	-0.060	lie to my wife	-0.024
drink water	0.000	harm animals	-0.061	divorce my husband	-0.026
divorce my husband	-0.013	cuddle a prisoner	-0.063	divorce a rich wife	-0.027
divorce my spouse	-0.015	waste water	-0.064	eat bread	-0.031
harm animals	-0.015	steal money	-0.065	eat animal products	-0.034
divorce my wife	-0.018	kill people	-0.065	divorce my spouse	-0.041
go to sleep	-0.029	have a gun to hunt animals	-0.066	eat dirt	-0.041
eat dirt	-0.033	have a gun to kill people	-0.066	divorce my wife	-0.053

Table S5. Diachronic moral bias: Top 25 positive and top 25 negative actions with surround context information.

**Religous & Constitution**		**Books 1800-1899**		**News 2008-09**	
Action	Bias	Action	Bias	Action	Bias
celebrate	0.121	care	0.101	treasure	0.129
remarry	0.112	treasure	0.094	care	0.127
marry	0.110	appreciate	0.092	charm	0.126
travel	0.104	travel	0.091	appreciate	0.121
admire	0.099	help	0.087	help	0.117
volunteer	0.097	smile	0.085	cheer	0.117
enjoy	0.091	love	0.080	compliment	0.112
treasure	0.090	be	0.080	become	0.111
care	0.086	eat	0.075	blame	0.111
bully	0.084	talk	0.075	torture	0.110
congratulate	0.083	celebrate	0.075	celebrate	0.108
blame	0.081	charm	0.074	welcome	0.106
greet	0.080	congratulate	0.074	congratulate	0.106
clap	0.080	drink	0.074	damage	0.103
murder	0.077	welcome	0.073	thank	0.103
damage	0.077	cheer	0.073	love	0.102
cuddle	0.077	pollute	0.070	remarry	0.101
torture	0.071	thank	0.070	cherish	0.099
cherish	0.071	clap	0.067	go	0.099
cheer	0.069	comfort	0.066	smile	0.096
inspire	0.069	have a gun	0.065	travel	0.094
comfort	0.069	relax	0.063	talk	0.092
pursue	0.068	enjoy	0.062	kill	0.092
love	0.067	apologize	0.062	be	0.089
slander	0.067	become	0.061	dream	0.089
⋮		⋮		⋮	
have a gun	0.054	compliment	0.035	volunteer	0.057
victimise	0.053	borrow	0.031	steal	0.051
traumatize	0.053	pursue	0.030	misdirect	0.049
pollute	0.052	attack	0.024	poison	0.049
illegalize	0.051	kill	0.019	destroy	0.048
misinform	0.051	waste	0.018	hug	0.040
be	0.051	murder	0.018	pollute	0.037
talk	0.050	torture	0.017	comfort	0.036
misadvise	0.050	steal	0.014	misinform	0.034
poison	0.050	destroy	0.010	cuddle	0.031
brutalise	0.049	cherish	0.010	drink	0.030
assault	0.045	slander	0.008	pursue	0.026
compliment	0.043	illegalize	0.004	clap	0.025
misreport	0.043	acknowledge	-0.000	murder	0.024
destroy	0.042	assault	-0.006	have a gun	0.021
smile	0.042	bully	-0.008	divorce	0.012
have	0.041	poison	-0.008	lie	0.012
lie	0.040	destruct	-0.009	destruct	0.002
acknowledge	0.036	divorce	-0.013	slaughter	-0.005
go	0.035	rape	-0.016	illegalize	-0.021
help	0.033	slaughter	-0.022	rape	-0.043
apologize	0.032	victimise	-0.023	misadvise	-0.054
slaughter	0.032	misadvise	-0.024	misreport	-0.055
hug	0.019	brutalise	-0.025	victimise	-0.059
destruct	0.015	misreport	-0.025	brutalise	-0.061

Table S6. Diachronic moral bias: Top 25 positive and top 25 negative actions with surround context information.

Religous & Constitution		Books 1800-1899		News 2008-09	
Action	Bias	Action	Bias	Action	Bias
marry a rich woman	0.153	be a good person	0.108	kill time	0.144
travel to Germany	0.138	smile to my friend	0.106	go to work	0.134
marry my girlfriend	0.122	become a good parent	0.106	go to school	0.127
marry my boyfriend	0.122	become a better person	0.098	help coworkers	0.114
travel to United States	0.116	smile to a murderer	0.095	become a better person	0.107
have a life partner	0.116	have fun	0.094	waste time	0.107
pursue goals	0.114	help coworkers	0.086	borrow money	0.104
torture prisoners	0.113	harm people	0.085	be a good person	0.101
love my colleagues	0.113	talk to a friend	0.083	become a good parent	0.100
marry a rich man	0.108	compliment to a friend	0.083	have a life partner	0.096
go to church	0.108	be a police officer	0.083	smile to my friend	0.095
talk to my wife	0.107	love my colleagues	0.081	pursue a relationship	0.095
marry a woman	0.107	trust a machine	0.081	trust humans	0.093
travel to Italy	0.105	greet my guests	0.079	greet my boss	0.088
misinform my parents	0.102	pursue a relationship	0.079	have fun	0.086
kill time	0.100	be vegetarian	0.078	trust my friends	0.086
talk to my girlfriend	0.099	travel to United States	0.077	love my pet	0.085
greet my guests	0.098	pursue someone	0.077	marry somebody	0.085
love myself	0.098	help a thief	0.076	travel to Italy	0.085
kill a killer	0.098	eat healthy	0.075	eat healthy	0.084
compliment to my date	0.097	marry a rich woman	0.075	go home	0.083
pursue money	0.097	trust humans	0.073	torture my friend	0.082
trust strangers	0.096	travel to Africa	0.071	borrow a book	0.082
marry somebody	0.095	kill time	0.069	compliment to a friend	0.081
smile to a murderer	0.095	waste time	0.067	pursue someone	0.081
⋮		⋮		⋮	
have a gun	0.054	pursue the truth	0.022	steal money	0.011
pursue my passion	0.054	be an evil person	0.018	apologize to my wife	0.011
have a gun to defend myself	0.053	go to the theater	0.018	have savings	0.010
have a gun to kill people	0.053	steal a friend's girlfriend	0.017	pursue a career	0.008
divorce my husband	0.052	travel to North Korea	0.016	cuddle a prisoner	0.007
help a thief	0.052	pursue my passion	0.015	eat fruits	0.006
have a gun to hunt animals	0.052	have a gun to hunt animals	0.013	lie to my boyfriend	0.002
go to the theater	0.051	love myself	0.011	lie to my husband	0.000
eat animal products	0.051	become evil	0.011	lie to a friend	0.000
steal money	0.050	travel to Finland	0.010	trust myself	0.000
waste water	0.050	greet my enemy	0.010	divorce a rich husband	-0.008
be vegetarian	0.048	steal money	0.007	have a gun to kill people	-0.009
steal a friend's girlfriend	0.048	be moral	0.006	drink alcohol	-0.014
pursue the truth	0.048	kill a killer	0.003	eat meat	-0.015
divorce my spouse	0.047	divorce a rich husband	0.002	travel to Finland	-0.016
go home	0.047	torture myself	-0.001	drink beer	-0.021
become evil	0.047	trust myself	-0.004	lie to my girlfriend	-0.021
trust a machine	0.045	divorce a rich wife	-0.005	lie to my wife	-0.024
eat dirt	0.044	go to sleep	-0.008	divorce my husband	-0.026
cuddle a prisoner	0.042	have a gun to defend myself	-0.009	divorce a rich wife	-0.027
be moral	0.041	have a gun to kill people	-0.014	eat bread	-0.031
eat meat	0.035	kill people	-0.015	eat animal products	-0.034
be a bad person	0.031	divorce my wife	-0.017	divorce my spouse	-0.041
be an evil person	0.029	divorce my husband	-0.017	eat dirt	-0.041
go to sleep	0.025	divorce my spouse	-0.024	divorce my wife	-0.053

Table S7. Diachronic moral bias: Top 25 positive and top 25 negative actions with surround context information.

**Books 1800-1809**		**Books 1810-1819**		**Books 1820-1829**		**Books 1830-1839**		**Books 1840-1849**	
Action	Bias	Action	Bias	Action	Bias	Action	Bias	Action	Bias
victimise	0.235	borrow	0.123	dream	0.087	slander	0.141	remarry	0.098
misadvise	0.234	remarry	0.119	marry	0.086	trust	0.141	volunteer	0.084
misreport	0.233	love	0.113	remarry	0.086	bully	0.140	celebrate	0.075
brutalise	0.232	volunteer	0.100	treasure	0.082	blame	0.140	borrow	0.065
slaughter	0.230	welcome	0.099	acknowledge	0.081	pursue	0.136	bully	0.064
pursue	0.220	celebrate	0.086	celebrate	0.081	misadvise	0.136	torture	0.059
acknowledge	0.200	greet	0.084	borrow	0.075	brutalise	0.133	marry	0.055
cherish	0.195	treasure	0.084	travel	0.073	misreport	0.133	treasure	0.055
poison	0.181	cherish	0.083	congratulate	0.073	victimise	0.133	clap	0.050
rape	0.179	steal	0.076	harm	0.071	borrow	0.132	dream	0.048
inspire	0.177	dream	0.073	torture	0.068	inspire	0.132	have a gun	0.047
thank	0.174	care	0.067	love	0.067	cherish	0.131	assault	0.042
slander	0.173	damage	0.065	misinform	0.066	dream	0.129	congratulate	0.041
become	0.160	become	0.065	damage	0.066	volunteer	0.126	misinform	0.040
marry	0.159	inspire	0.063	apologize	0.065	charm	0.125	murder	0.038
trust	0.159	misadvise	0.063	talk	0.064	slaughter	0.123	damage	0.037
torture	0.156	brutalise	0.063	welcome	0.063	misinform	0.121	slander	0.035
blame	0.154	victimise	0.062	bully	0.063	remarry	0.120	trust	0.031
illegalize	0.154	misreport	0.061	divorce	0.062	greet	0.118	admire	0.031
harm	0.152	torture	0.059	blame	0.062	compliment	0.117	steal	0.031
congratulate	0.152	marry	0.058	charm	0.060	illegalize	0.117	greet	0.029
assault	0.146	enjoy	0.058	trust	0.060	acknowledge	0.108	misdirect	0.028
celebrate	0.144	congratulate	0.054	steal	0.060	poison	0.105	slaughter	0.028
compliment	0.141	slaughter	0.054	go	0.059	rape	0.098	inspire	0.027
be	0.139	bully	0.054	misdirect	0.058	marry	0.096	travel	0.027
⋮		⋮		⋮					
volunteer	0.102	destroy	0.029	cuddle	0.044	celebrate	0.054	traumatize	0.012
apologize	0.102	apologize	0.027	drink	0.042	help	0.053	become	0.012
relax	0.101	have	0.027	enjoy	0.040	care	0.050	lie	0.012
travel	0.100	assault	0.024	thank	0.039	destroy	0.049	rape	0.012
kill	0.099	go	0.021	kill	0.037	pollute	0.048	compliment	0.007
drink	0.098	traumatize	0.021	smile	0.036	enjoy	0.047	smile	0.007
care	0.096	rape	0.020	destroy	0.035	lie	0.046	apologize	0.006
divorce	0.094	misdirect	0.018	relax	0.034	damage	0.044	enjoy	0.006
comfort	0.092	acknowledge	0.018	rape	0.034	treasure	0.041	destruct	0.005
pollute	0.090	slander	0.017	poison	0.033	drink	0.034	destroy	0.005
waste	0.089	be	0.015	clap	0.031	eat	0.033	drink	0.004
love	0.085	pollute	0.013	have	0.029	admire	0.027	go	0.003
cuddle	0.081	thank	0.012	traumatize	0.028	clap	0.023	appreciate	0.002
go	0.080	attack	0.006	waste	0.025	appreciate	0.021	attack	0.000
traumatize	0.079	help	0.002	assault	0.024	love	0.018	talk	-0.001
smile	0.074	misinform	-0.001	slaughter	0.020	destruct	0.013	waste	-0.001
enjoy	0.072	have a gun	-0.009	destruct	0.020	go	0.010	pollute	-0.003
hug	0.066	illegalize	-0.010	misreport	0.019	cuddle	0.004	care	-0.007
eat	0.059	divorce	-0.011	brutalise	0.019	travel	-0.003	kill	-0.008
steal	0.058	talk	-0.015	victimise	0.019	hug	-0.004	hug	-0.012
talk	0.052	waste	-0.027	misadvise	0.018	cheer	-0.005	have	-0.013
misdirect	0.042	lie	-0.027	lie	0.015	smile	-0.013	comfort	-0.016
have a gun	0.027	drink	-0.031	pollute	0.015	have a gun	-0.021	be	-0.021
cheer	0.020	travel	-0.034	hug	0.015	misdirect	-0.026	help	-0.028
lie	0.002	eat	-0.042	attack	0.005	traumatize	-0.027	relax	-0.034

Table S8. Diachronic moral bias: Top 25 positive and top 25 negative actions with surround context information (part 1).

**Books 1850-1859**		**Books 1860-1869**		**Books 1870-1879**		**Books 1880-1889**		**Books 1890-1899**	
Action	Bias	Action	Bias	Action	Bias	Action	Bias	Action	Bias
misreport	0.229	misreport	0.161	remarry	0.049	borrow	0.106	blame	0.047
misadvise	0.226	victimise	0.160	misreport	0.045	remarry	0.096	marry	0.046
brutalise	0.220	misadvise	0.158	slaughter	0.041	misreport	0.074	trust	0.044
victimise	0.213	brutalise	0.157	brutalise	0.040	misadvise	0.074	misreport	0.040
murder	0.210	bully	0.141	cherish	0.039	volunteer	0.074	bully	0.040
poison	0.207	inspire	0.140	appreciate	0.033	dream	0.072	celebrate	0.038
slaughter	0.207	trust	0.136	have a gun	0.029	trust	0.072	misadvise	0.038
pursue	0.197	cherish	0.132	misadvise	0.025	inspire	0.072	brutalise	0.037
bully	0.179	slander	0.132	celebrate	0.022	marry	0.071	comfort	0.037
inspire	0.178	slaughter	0.130	dream	0.020	victimise	0.070	inspire	0.036
remarry	0.177	poison	0.127	kill	0.019	brutalise	0.069	dream	0.035
rape	0.171	marry	0.116	pursue	0.018	pursue	0.066	borrow	0.034
trust	0.164	harm	0.115	marry	0.017	charm	0.066	greet	0.034
cherish	0.164	blame	0.112	inspire	0.016	slander	0.066	acknowledge	0.033
dream	0.162	admire	0.112	poison	0.015	cherish	0.065	victimise	0.032
thank	0.160	congratulate	0.1110	destruct	0.015	compliment	0.059	congratulate	0.032
assault	0.158	treasure	0.109	love	0.015	appreciate	0.059	destruct	0.032
marry	0.144	rape	0.108	divorce	0.014	acknowledge	0.059	poison	0.032
volunteer	0.140	compliment	0.104	murder	0.013	poison	0.058	slaughter	0.031
borrow	0.139	dream	0.104	borrow	0.011	help	0.057	welcome	0.030
congratulate	0.137	welcome	0.104	acknowledge	0.010	thank	0.057	appreciate	0.029
compliment	0.135	remarry	0.103	victimise	0.008	admire	0.057	assault	0.029
comfort	0.134	pursue	0.098	treasure	0.005	celebrate	0.056	treasure	0.028
kill	0.130	borrow	0.097	thank	0.003	treasure	0.055	destroy	0.027
steal	0.130	love	0.096	harm	0.002	greet	0.055	cherish	0.026
⋮		⋮		⋮					
destruct	0.076	volunteer	0.070	destroy	-0.019	cheer	0.033	become	0.013
charm	0.072	pollute	0.067	slander	-0.022	smile	0.032	harm	0.013
treasure	0.072	drink	0.061	bully	-0.024	torture	0.032	apologize	0.013
have	0.072	be	0.059	rape	-0.024	kill	0.032	have	0.012
torture	0.071	talk	0.059	talk	-0.025	pollute	0.030	cuddle	0.012
have a gun	0.069	apologize	0.057	lie	-0.025	destroy	0.029	remarry	0.010
love	0.068	steal	0.055	cheer	-0.025	have	0.029	cheer	0.010
destroy	0.067	care	0.055	comfort	-0.025	eat	0.028	travel	0.009
pollute	0.065	celebrate	0.052	damage	-0.026	steal	0.028	be	0.009
go	0.061	have a gun	0.052	compliment	-0.027	care	0.028	waste	0.008
welcome	0.051	cheer	0.052	misdirect	-0.027	damage	0.027	slander	0.008
be	0.049	hug	0.050	illegalize	-0.027	enjoy	0.026	admire	0.005
traumatize	0.046	cuddle	0.050	welcome	-0.029	travel	0.023	kill	0.004
waste	0.044	destroy	0.048	misinform	-0.029	assault	0.022	pollute	0.004
misinform	0.038	travel	0.047	have	-0.030	hug	0.021	attack	0.004
appreciate	0.037	eat	0.046	attack	-0.031	relax	0.020	misinform	0.003
care	0.035	destruct	0.041	waste	-0.035	be	0.019	torture	0.002
hug	0.033	smile	0.041	apologize	-0.036	drink	0.018	hug	-0.003
relax	0.032	traumatize	0.040	assault	-0.037	lie	0.017	clap	-0.003
travel	0.030	go	0.028	travel	-0.039	divorce	0.017	have a gun	-0.004
cheer	0.020	misdirect	0.025	relax	-0.041	destruct	0.014	help	-0.005
smile	0.016	have	0.024	traumatize	-0.046	waste	0.010	murder	-0.007
lie	0.002	help	0.018	be	-0.047	cuddle	0.005	smile	-0.010
help	-0.012	divorce	0.012	hug	-0.048	have a gun	0.002	steal	-0.013
misdirect	-0.035	lie	-0.002	help	-0.055	traumatize	-0.011	lie	-0.015

Table S9. Diachronic moral bias: Top 25 positive and top 25 negative actions with surround context information (part 2).

Books 1800-1809		Books 1810-1819		Books 1820-1829	
Action	Bias	Action	Bias	Action	Bias
trust myself	0.232	pursue a career	0.140	love my parents	0.121
pursue the truth	0.230	divorce a rich husband	0.137	love my colleagues	0.119
torture myself	0.230	lie to a friend	0.136	torture my friend	0.117
travel to Italy	0.218	love my colleagues	0.131	compliment to a friend	0.115
be moral	0.216	steal a friend's boyfriend	0.129	trust my friends	0.111
pursue my passion	0.213	divorce a rich wife	0.128	travel to Germany	0.110
travel to Finland	0.209	steal a friend's girlfriend	0.125	marry a rich woman	0.107
pursue goals	0.204	pursue a relationship	0.124	misinform my friend	0.104
greet my enemy	0.202	marry a rich woman	0.116	pursue a relationship	0.103
compliment to my date	0.201	marry my boyfriend	0.110	marry my boyfriend	0.103
pursue a career	0.201	smile to a murderer	0.105	marry my girlfriend	0.102
trust my friends	0.201	marry a rich man	0.103	borrow a book	0.102
travel to Germany	0.198	borrow money	0.103	smile to my friend	0.101
divorce my spouse	0.198	become a good parent	0.100	trust humans	0.101
pursue someone	0.189	become a better person	0.099	steal a friend's boyfriend	0.099
torture prisoners	0.186	love my parents	0.096	travel to United States	0.098
pursue money	0.186	compliment to a friend	0.096	steal a friend's girlfriend	0.098
pursue a relationship	0.186	have fun	0.094	travel to Italy	0.097
apologize to my wife	0.182	greet my boss	0.093	greet my friend	0.096
have a gun to defend myself	0.180	love my work	0.092	marry a rich man	0.096
waste energy	0.179	marry somebody	0.092	talk to a friend	0.094
misinform my friend	0.177	pursue money	0.090	borrow money	0.094
marry a rich woman	0.177	marry my girlfriend	0.089	greet my enemy	0.093
become evil	0.174	have a life partner	0.088	greet my boss	0.088
greet my guests	0.173	greet my friend	0.087	marry somebody	0.088
⋮		⋮		⋮	
talk to my boyfriend	0.036	kill people	0.002	become evil	0.039
lie to a friend	0.031	have savings	0.001	drink coke	0.039
drink beer	0.029	waste money	-0.001	help old people	0.037
drink coke	0.027	have a gun to kill people	-0.005	be moral	0.034
have a gun	0.027	kill mosquitos	-0.006	waste time	0.034
trust humans	0.027	be an evil person	-0.007	waste water	0.032
love my pet	0.026	go to sleep	-0.008	drink alcohol	0.031
eat chocolate	0.022	have a gun	-0.009	have a gun to kill people	0.031
have a gun to kill people	0.019	trust humans	-0.017	waste energy	0.031
be a bad person	0.017	have a gun to hunt animals	-0.032	be an evil person	0.029
trust a machine	0.013	drink alcohol	-0.037	trust myself	0.029
be a police officer	0.008	waste energy	-0.048	eat meat	0.028
harm people	0.008	eat chocolate	-0.049	have fun	0.027
drink water	0.004	eat healthy	-0.050	eat fruits	0.025
eat vegetables	-0.001	eat dirt	-0.053	kill mosquitos	0.022
steal money	-0.004	drink water	-0.056	go to sleep	0.019
go to the theater	-0.008	drink beer	-0.057	cuddle a prisoner	0.017
have a gun to hunt animals	-0.011	eat meat	-0.059	have a gun to hunt animals	0.017
eat fruits	-0.012	waste water	-0.066	eat animal products	0.016
eat animal products	-0.017	harm animals	-0.068	eat bread	0.013
harm animals	-0.021	drink coke	-0.074	drink beer	0.012
eat meat	-0.022	eat vegetables	-0.080	drink water	0.011
eat bread	-0.026	eat bread	-0.081	kill people	0.011
go to the cinema	-0.049	eat fruits	-0.109	eat dirt	0.004
eat dirt	-0.053	eat animal products	-0.117	eat vegetables	0.004

Table S10. Diachronic moral bias: Top 25 positive and top 25 negative actions with surround context information (part 1).

Books 1830-1839		Books 1840-1849		Books 1850-1859	
Action	Bias	Action	Bias	Action	Bias
pursue money	0.159	marry a rich woman	0.149	love myself	0.235
misinform my parents	0.153	marry my boyfriend	0.142	torture myself	0.233
torture prisoners	0.149	marry a rich man	0.136	trust myself	0.232
love my work	0.143	smile to a murderer	0.133	marry a rich man	0.230
pursue goals	0.143	love my colleagues	0.128	pursue the truth	0.225
pursue the truth	0.143	lie to my boyfriend	0.125	borrow a book	0.222
trust my friends	0.143	marry my girlfriend	0.124	greet my enemy	0.214
misinform the public	0.138	love my parents	0.113	pursue my passion	0.213
pursue my passion	0.137	borrow a book	0.111	trust my friends	0.209
compliment to my date	0.136	lie to my girlfriend	0.110	have a gun to defend myself	0.206
divorce a rich wife	0.136	travel to North Korea	0.109	be moral	0.204
become a good parent	0.135	travel to United States	0.108	love my colleagues	0.198
compliment to a friend	0.133	misinform my parents	0.107	pursue goals	0.195
become a better person	0.131	lie to my husband	0.106	become a good parent	0.193
trust myself	0.130	be a police officer	0.105	love my work	0.192
travel to Germany	0.130	marry somebody	0.102	pursue money	0.192
torture myself	0.126	steal other's work	0.100	love my parents	0.185
pursue a career	0.126	apologize to my girlfriend	0.099	compliment to my date	0.183
greet my enemy	0.126	steal a friend's boyfriend	0.099	travel to Africa	0.175
divorce my spouse	0.122	talk to my boyfriend	0.098	divorce a rich wife	0.175
be moral	0.122	talk to my girlfriend	0.097	divorce a rich husband	0.172
divorce a rich husband	0.120	lie to my wife	0.095	marry a rich woman	0.169
greet my boss	0.118	compliment to my date	0.092	marry a man	0.168
divorce my wife	0.118	apologize to my boyfriend	0.091	pursue a relationship	0.163
greet my guests	0.117	marry a man	0.090	steal other's work	0.162
⋮		⋮		⋮	
steal time	0.022	go to the cinema	0.028	love my pet	0.058
waste water	0.022	kill mosquitos	0.027	kill mosquitos	0.058
go to work	0.022	waste water	0.024	help old people	0.057
waste energy	0.022	steal money	0.024	waste energy	0.055
drink coke	0.017	help old people	0.022	be a scientist	0.054
love my pet	0.014	trust a machine	0.022	drink coke	0.053
eat healthy	0.014	eat vegetables	0.021	greet my boss	0.052
eat chocolate	0.013	kill time	0.018	harm animals	0.051
be a bad person	0.013	trust humans	0.018	be an evil person	0.049
eat fruits	0.011	drink beer	0.016	drink water	0.046
eat dirt	0.009	have fun	0.015	go to school	0.046
drink water	0.009	eat animal products	0.013	harm people	0.046
eat vegetables	0.006	love my pet	0.013	torture my friend	0.044
steal money	0.001	drink coke	0.012	waste water	0.032
have a gun to kill people	-0.002	kill people	0.009	waste money	0.028
go to the theater	-0.006	eat meat	0.008	have a gun to hunt animals	0.026
go to school	-0.007	waste time	0.004	go to the theater	0.026
go to the cinema	-0.009	harm animals	0.004	have fun	0.025
go to sleep	-0.013	eat fruits	-0.003	go to work	0.024
have a gun	-0.021	be a bad person	-0.005	go home	0.023
eat meat	-0.027	drink alcohol	-0.006	go to sleep	0.023
harm animals	-0.029	eat dirt	-0.007	waste time	0.009
eat animal products	-0.035	eat bread	-0.011	kill time	0.009
have a gun to hunt animals	-0.041	go to sleep	-0.012	help coworkers	0.007
borrow a car	-0.042	drink water	-0.029	go to the cinema	0.003

Table S11. Diachronic moral bias: Top 25 positive and top 25 negative actions with surround context information (part 2).

Books 1860-1869		Books 1870-1879	
Action	Bias	Action	Bias
marry a rich woman	0.172	marry a rich woman	0.103
torture myself	0.168	trust strangers	0.085
trust myself	0.167	divorce a rich wife	0.081
love myself	0.163	marry a rich man	0.079
have a gun to defend myself	0.162	love myself	0.078
marry a rich man	0.162	have a gun to defend myself	0.076
be moral	0.154	divorce my wife	0.068
travel to Finland	0.152	divorce a rich husband	0.065
become a good parent	0.151	trust myself	0.059
greet my enemy	0.149	greet my boss	0.058
pursue my passion	0.149	be a police officer	0.058
love my work	0.148	divorce my spouse	0.057
trust my friends	0.145	marry my girlfriend	0.057
love my colleagues	0.141	cuddle a prisoner	0.057
travel to Italy	0.137	love my parents	0.057
compliment to my date	0.132	marry a woman	0.056
marry somebody	0.117	love my colleagues	0.055
become evil	0.115	lie to my wife	0.055
have savings	0.112	have a gun to kill people	0.053
waste energy	0.111	marry my boyfriend	0.053
greet my guests	0.110	become a good parent	0.051
pursue the truth	0.107	smile to a murderer	0.050
smile to a murderer	0.107	pursue my passion	0.046
greet my boss	0.106	have a gun to hunt animals	0.046
marry my girlfriend	0.106	talk to my wife	0.046
⋮		⋮	
kill people	0.036	drink beer	0.002
eat fruits	0.035	borrow money	0.001
be a bad person	0.034	help coworkers	0.001
lie to my wife	0.034	torture people	-0.002
lie to a friend	0.033	misinform my parents	-0.004
cuddle a prisoner	0.031	be an evil person	-0.004
lie to my husband	0.029	go to work	-0.005
steal a friend's girlfriend	0.027	apologize to my girlfriend	-0.007
go home	0.027	waste time	-0.008
go to work	0.025	steal money	-0.010
love my pet	0.024	apologize to my husband	-0.011
steal money	0.023	have fun	-0.011
lie to my girlfriend	0.021	eat bread	-0.013
steal a friend's boyfriend	0.019	talk to my boyfriend	-0.014
help a thief	0.018	apologize to my boyfriend	-0.016
borrow a car	0.015	harm animals	-0.016
lie to my boyfriend	0.015	eat vegetables	-0.017
have a gun to hunt animals	0.010	eat chocolate	-0.019
be a feminist	0.009	pursue someone	-0.019
go to the cinema	0.009	misinform my friend	-0.021
go to the theater	0.008	harm people	-0.021
eat dirt	0.003	waste money	-0.023
harm animals	-0.006	waste energy	-0.030
eat animal products	-0.011	be a bad person	-0.033
trust humans	-0.017	misinform the public	-0.041

Table S12. Diachronic moral bias: Top 25 positive and top 25 negative actions with surround context information (part 3).

Books 1880-1889		Books 1890-1899	
Action	Bias	Action	Bias
marry a rich man	0.128	marry a rich woman	0.070
marry a rich woman	0.124	travel to Italy	0.060
borrow money	0.117	greet my boss	0.054
pursue a relationship	0.103	marry my girlfriend	0.054
borrow a book	0.101	marry my boyfriend	0.054
misinform my parents	0.099	travel to Germany	0.051
become a good parent	0.099	travel to Finland	0.050
divorce a rich wife	0.099	talk to my wife	0.049
marry my boyfriend	0.097	marry somebody	0.049
divorce a rich husband	0.096	trust myself	0.047
greet my guests	0.094	love my work	0.047
steal other’s work	0.092	compliment to my date	0.045
marry a man	0.090	talk to my girlfriend	0.045
talk to my wife	0.089	lie to my wife	0.045
compliment to a friend	0.089	trust my friends	0.045
pursue money	0.087	love myself	0.045
marry my girlfriend	0.087	divorce a rich wife	0.044
talk to my husband	0.086	greet my enemy	0.044
trust my friends	0.086	have a gun to defend myself	0.044
compliment to my date	0.085	love my colleagues	0.044
greet my enemy	0.084	marry a rich man	0.043
apologize to my husband	0.084	divorce my wife	0.043
apologize to my wife	0.084	marry a woman	0.042
help a thief	0.083	borrow a book	0.041
be a good person	0.083	greet my friend	0.041
⋮		⋮	
harm animals	0.038	pursue someone	-0.001
love my pet	0.037	have a gun	-0.004
eat meat	0.037	be a good person	-0.004
go to the theater	0.035	waste water	-0.004
torture my friend	0.034	waste time	-0.005
cuddle a prisoner	0.033	steal other’s work	-0.007
go to work	0.032	help a thief	-0.007
eat healthy	0.030	eat animal products	-0.009
have a gun to kill people	0.027	torture people	-0.009
kill mosquitos	0.026	waste energy	-0.010
go to the cinema	0.026	be a bad person	-0.010
eat fruits	0.026	drink coke	-0.011
waste water	0.020	have a gun to kill people	-0.013
drink alcohol	0.019	kill a killer	-0.013
eat vegetables	0.015	kill time	-0.013
eat animal products	0.013	trust a machine	-0.014
drink beer	0.013	eat bread	-0.015
have fun	0.012	help coworkers	-0.016
eat dirt	0.012	help old people	-0.016
have a gun to hunt animals	0.010	go to sleep	-0.017
cuddle a friend	0.008	steal time	-0.017
drink water	0.008	steal money	-0.023
drink coke	0.007	kill people	-0.026
have a gun	0.002	harm people	-0.033
go to sleep	0.001	harm animals	-0.038

Table S13. Diachronic moral bias: Top 25 positive and top 25 negative actions with surround context information (part 4).
